# Determining the influence of variable additive, filler, and dye concentrations in plastics on their fluorescence behavior via spectrometry and FD-FLIM

**DOI:** 10.1007/s00216-026-06361-0

**Published:** 2026-02-05

**Authors:** Maximilian Wohlschläger, Markus Bonauer, Manuela List, Martin Versen, Martin G. J. Löder, Christian Laforsch

**Affiliations:** 1 Faculty of Engineering Sciences, Rosenheim Technical University of Applied Sciences, Hochschulstraße 1, 83024 Rosenheim, Germany; 2 Faculty of Chemical Technology and Economics, Rosenheim Technical University of Applied Sciences (Campus Burghausen), Robert-Koch-Straße 28, 84489 Burghausen, Germany; 3 Animal Ecology I and BayCEER, University Bayreuth, Universitätsstraße 30, 95440 Bayreuth, Germany

**Keywords:** Microplastic contamination, Plastic identification, Additive concentration dependency, Fluorescence lifetime, FD-FLIM

## Abstract

Microplastic (MP) pollutes our terrestrial and aquatic ecosystems due to their uncontrolled discharge into our environment. The analysis of MP contamination is still a challenge, although significant improvements are made for different environmental matrices. Using mass-based particle analysis methods such as thermal extraction and desorption-gas chromatography/mass spectroscopy (GC/MS) or pyrolysis GC/MS, essential parameters such as the MP’s morphology, size, and shape cannot be obtained, which are indispensable to assess the hazard of the respective particles. Raman, micro-Fourier transform infrared, and attenuated total reflectance spectroscopy are particle-based analysis methods, which are time-consuming due to the high purification effort. Thus, novel, reliable, and time-efficient methods for MP analysis are required. Previously, studies showed the potential of frequency domain fluorescence lifetime imaging microscopy (FD-FLIM) to identify plastics’ type, shape, size, and morphology, and distinguishing these from natural materials. However, only pure plastic granules were investigated, omitting that commodity plastics accumulating in our environment contain various additive, filler, or dye concentrations. To circumvent the dependency of additive, filler, and dye concentrations, we investigated the fluorescence spectra and lifetimes of three plastic types, individually composed with two fillers, three additives, and two dyes in six different concentrations. We heuristically modeled the dependency of the concentration on plastics’ fluorescence lifetime using a logarithmic model with a high correlation and showed that identifying the plastic types is hardly possible when fillers, additives, or dyes are added in various concentrations because of their superimposing fluorescence lifetimes. However, further research has to be conducted to investigate different emission states of fluorescence to optimize the FD-FLIM method, as only one excitation wavelength and emission band was used for the investigations.

**Supplementary Information:**

The online version contains supplementary material available at 10.1007/s00216-026-06361-0.

## Introduction

The pollution of our environment by micro- and nanoplastics (MP and NP) is increasing yearly due to the uncontrolled discharge of millions of tons of plastic waste [1]. Furthermore, MNP can directly be released into the environment by different sources via abrasion of e.g. tire wear, dispersion paints, textiles, or geotextiles or by the application of e.g. plastic encapsulated fertilizers or plastic-coated seeds [2–5]. This increase in pollution of our terrestrial and aquatic ecosystems poses a threat to environmental and human health [6–12]. Hereby, the hazard depends on several factors, such as the level of exposure, exposure time of the organism to the plastic, and the plastic particle properties, such as plastic type, shape, size, and morphology [3]. To accurately and rapidly obtain these properties, along with the level of contamination, will foster hazard assessment for this diverse suite of contaminants.

Mass-based and particle-based chemical identification methods have prevailed in determining the plastics’ properties. To analyze the mass proportion of MPs in an environmental sample, thermal extraction and desorption-gas chromatography/mass spectroscopy (TED-GC/MS) [13, 14] or pyrolysis GC/MS [15, 16] are used. However, these mass-based methods cannot be used to determine particle properties such as morphology, shape, and size, as well as the number of MP particles in the sample, which are essential to properly assess the hazard posed by MP.

The primary particle-based analysis methods of MPs are Raman, micro-Fourier transform infrared (FTIR), or attenuated total reflectance (ATR) FTIR spectroscopy. Although plenty of essential MP properties can be obtained using these spectroscopic methods, sample extraction and purification, identification, and quantification are very time-consuming [10]. Especially the step of sample extraction and purification must be done carefully as the spectroscopic analysis only gives the desired reliable results on MP particles if they are immaculate and anhydrous [17]. Consequently, it is evident that directly identifying MPs in environmental samples using these spectroscopic methods is challenging [18]. Other research shows that particle-based detection of microplastics in freshwater is possible using ultra-high-definition (UHD) optical imaging [19]. However, this UHD imaging approach relies solely on contrast and particle morphology, which means that the chemical composition of the plastic types cannot be obtained and therefore the toxicity of the particles cannot be determined. As a result, a high-throughput analysis of MPs in environmental samples is not possible using particle-based analysis methods.

Directly identifying MPs in an environmental sample would circumvent the issues of mass- and particle-based analysis methods and positively impact the efficiency of determining the contamination level. Additionally, this would yield a substantial increase in sample throughput and have a positive effect on MP monitoring programs. Nevertheless, methods to directly identify MPs in terrestrial or aquatic environments are currently unavailable or under development.

The doping of plastics with fluorescent markers to identify them using their fluorescence spectra was previously highlighted [20, 21]. However, this approach is only applicable if plastics are doped with maybe toxic fluorescence markers in a time-consuming pre-treatment. Further, characteristic autofluorescence exhibited by plastics and MP has been suggested, as the fluorescence behavior of different plastic types can be used for identification. In general, plastic types such as polyethylene (PE) or polypropylene (PP) should not exhibit any fluorescence emission, as they do not contain low-lying chromophores. Nevertheless, as chain breaks and oxidation products from manufacturing are present in commodity plastics, a fluorescence emission can be detected if excited by an intense laser pulse [22–24]. Therefore, plastics such as PP and PE, which have been improperly disposed of in the environment, should also exhibit fluorescence and thus a material characteristic fluorescence lifetime.

Fluorescence lifetimes can be determined in the time domain (TD) and frequency domain (FD) [25]. Langhals et al. and Gies et al. have previously shown that TD fluorescence lifetime could be used to identify plastic types, shape, size, and morphology [26, 27] and differentiate them from natural materials from aquatic environments [24]. In addition, Wohlschläger et al. reported that plastic type, shape, size, and morphology can also be identified on soil between natural materials at least down to a size of 70 µm in a single fluorescence lifetime image with FD fluorescence lifetime imaging microscopy (FD-FLIM) [28]. Additionally, identifying and differentiating plastics and natural materials from terrestrial environments is possible using FD-FLIM [29]. Furthermore, those materials can be classified via FD-FLIM in combination with neural networks [30]. FD-FLIM and TD-FLIM have advantages and disadvantages when evaluated against each other. TD-FLIM is more sensitive as only a single fluorescence wavelength is used when measuring the fluorescence lifetime. However, on the other hand, it takes more time to complete the measurement. The fluorescence lifetime measurement in FD-FLIM is either done with an optical band-pass or long-pass filter, which is slightly insensitive but results in a faster measurement.

Although TD-FLIM and FD-FLIM theoretically showed the potential for identifying MPs in an environmental matrix and differentiating MPs from environmental soil materials, only pure plastics were used in previous investigations [24, 26, 27]. However, commodity plastics in our environment contain different types of fillers, additives, and dyes in various concentrations, whereby their impact on the identifiability of the plastic type utilizing their fluorescence lifetime has yet to be researched, which is the major aim of this study. A previous investigation [31] showed that the fluorescence lifetime of polypropylene (PP) is impacted by a concentration of CaCO_3_, whereby an exponential increase in fluorescence lifetime with rising filler concentration was found. Nevertheless, as this was only a pilot study, it is unknown if and how different additive, filler, and dye concentrations affect the fluorescence lifetime and, if affected, whether it can be heuristically modeled or not, which is indispensable for making a direct identification of MPs in environmental samples possible.

In the present study, we aimed to answer two primary research questions: (I) Can the dependence of the fluorescence lifetime on filler, additive, and dye concentrations be heuristically modeled? and (II) Is the identification of the plastic type still possible via FD-FLIM, although additive, filler, and dye concentrations are added? To address these questions, we investigated the fluorescence spectra and lifetimes of three plastic types: low-density polyethylene (LDPE), polystyrene (PS), and PP containing different concentrations of two fillers (CaCO_3_, talcum), three additives (IRGANOX 1010, IRGAFOS 126, BYK-MAX LS 4125), and two dyes (PV Fast Red D3G, PV Fast Blue BG-IN) via FD-FLIM at 445 nm excitation. Based on the results, we heuristically modeled the dependence of fluorescence lifetime on filler, additive, and dye concentrations in plastics to provide information on the identifiability of plastic composites in environmental matrices.

## Theory

### Fluorescence lifetime measurements and fluorescence lifetime imaging

**Fig. 1:**
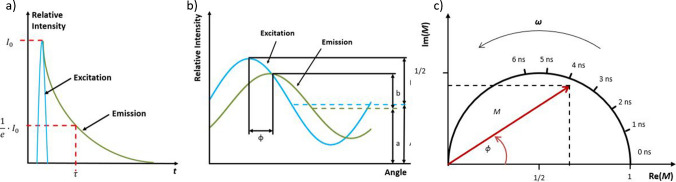
**a** Schematic representation of the time domain fluorescence measurement, incorporating the pulsed fluorescence excitation (blue) and the exponentially decreasing fluorescence (green). **b** Method of frequency domain fluorescence measurements, with a sinusoidal excitation signal (blue), causing the harmonic, phase-shifted, amplitude-damped, and equivalent shifted fluorescence emission (green). **c** Principle of the phasor plot, showing *M* as the length of the vector rotated around the origin by the phase shift *ϕ*, displayed in a unit circle, which is normalized by the modulation frequency *ω* from which a first indication of the fluorescence lifetime is given [28]

According to Lakowicz [25], the fluorescence lifetime can be determined in the time domain or frequency domain. Generally, the fluorescence lifetime *τ* is when the maximum fluorescence intensity *I*_0_ has decreased to *I*_0_/*e* (see Fig. 1a). The fluorescence lifetime is material-specific and lasts only a few nanoseconds. Using a time domain fluorescence lifetime measurement setup, the material is excited by a short laser pulse of defined wavelength (see Fig. 1a, blue curve), which causes a Stokes-shifted fluorescence emission with a maximum intensity *I*_0_, decaying exponentially after the excitation pulse ended (see Fig. 1a, green line). In the frequency domain, the fluorescence is caused by the Fourier transformation of the laser pulse, a sinusoidally modulated excitation signal. The modulated excitation signal has a defined modulation frequency *ω* (see Fig. 1b, blue oscillation curve), whereby the fluorescence emission follows this signal with an equal frequency. Nevertheless, as the fluorescence emission is lower in energy, the fluorescence signal is phase shifted by the angle *ϕ*, damped in its amplitude *a*, and shifted in its equivalent *b* concerning the excitation (*B*, *A*) (see Fig. 1b, green oscillation). By measuring the parameters of the phase shift *ϕ*, the amplitude damping from *A* to *a*, and the shift of the average value from *B* to *b*, a modulation index *M* and two fluorescence lifetimes: the phase-dependent fluorescence lifetime *τ*_*PH*_ and modulation-dependent fluorescence lifetime ${\tau }_{M}$ can be calculated, using Eqs. (1) – (3).

1 $${\tau }_{PH}=\frac{\mathrm{tan}\left(\phi \right)}{\omega }$$

2 $$M=\frac{(b/a)}{(B/A)}$$

3 $${\tau }_{M}=\frac{\sqrt{\frac{1}{{M}^{2}}-1}}{\omega }$$

Furthermore, in frequency domain fluorimetry, a representation of the modulation index and phase shift is possible in a phasor plot, shown schematically in Fig. 1c. The phasor plot combines the two measured core quantities of the fluorescence decay time measurement in the frequency range: the modulation index *M*, which represents the length of the arrow starting from the origin; and the phase shift *ϕ*, which represents the rotation of *M* around the origin.

FD-FLIM is based on the principles of measuring the fluorescence lifetime in the frequency domain. An imaging device measures the location-dependent sinusoidal or rectangular fluorescence emission. Calculating the location-dependent *ϕ*,* M*, and the corresponding fluorescence lifetimes ${\tau }_{PH}$ and ${\tau }_{M}$ is possible using the measured location-dependent fluorescence emission. Thus, an FD-FLIM measurement results in an image stack of five images: fluorescence intensity *I*, phase shift *ϕ*, modulation index *M*, phase-dependent fluorescence lifetime ${\tau }_{\mathrm{PH}}$, and modulation-dependent fluorescence lifetime ${\tau }_{M}$. Additionally, the phasor plot can be directly extracted, representing each pixel separately.

### Theoretical model

Initially, the fluorescence lifetime $\tau$ of a material can be described by the Strickler-Berg relation [25]. Nevertheless, Langhals and Schlücker stated that in the Strickler-Berg relation, no concentration dependency is included [32]. To provide evidence that the fluorescence lifetime is concentration-dependent, Langhals and Schlücker investigated the concentration dependency of a perylene fluorescence dye in chloroform on the fluorescence lifetime. With this, it was shown that the model in Eq. (4) fits the measured fluorescence lifetimes in dependence on the concentration for concentrations of 10^–6^ mol × L^−1^. Additionally, different concentrations of Nile Blue sulfate in ethanol were investigated to approve Eq. (4). As Langhals and Schlücker described, the parameters of the equation can be interpreted as follows: *a* describes the sensitivity of transposing the original linear to a logarithmic relationship; *C* is the concentration in [mol × L^−1^]; C* is the constant which characterizes the transposition from a linear to a logarithmic relation; and ${\tau }_{c= 0}$ is meant to be the fluorescence lifetime at an infinite small concentration.

As we already experienced in a previous investigation [31], that the fluorescence lifetime of PP is affected if different filler concentrations are added, we want to determine which influence different additive, filler, and dye concentrations have on the fluorescence lifetime of a plastic type and if the plastic type can be identified by heuristically modeling the dependency. To test if Eq. (4) can be used to answer our research questions, which influences different filler, additive, and dye concentrations have on the fluorescence behavior of different plastic types, we modified the equation such that the concentration *C* and concentration constant C* are given in *wt*%.

4 $$\tau =a\left[ns\right]*\mathrm{ln}\left(\frac{C[wt\%]}{{C}^{*}[wt\%]}+1\right)+{\tau }_{c= 0}[ns]$$

## Materials and methods

### Sample preparation using different additive, filler, and dye concentrations

To investigate if the plastic type can still be identified when additives, fillers, or dyes are added to the plastic, 126 samples containing variable concentrations of additives, fillers, or dyes were produced. Therefore, the plastics and different fillers, additives, and dyes were melted and homogenized in the micro compounder MC15HT (Xplore) and injection molded (IM) using the Micro-Injection-Moulder IM12 (Xplore). The resulting samples had standardized dog bone–shaped tensile bars. Additionally, three samples of pure plastics were produced to determine the influence of manufacturing on the fluorescence lifetime of the plastic. The base materials were the three plastic types: PP (PP Moplen HP500N P9T), PS (Styrolution PS 124 N), and LDPE (LUPOLEN 1800S). CaCO_3_ (Salt) and talcum (silicate) were used as fillers; IRGANOX 1010 (IRGANOX, antioxidant stabilizer; BASF), IRGAFOS 126 (IRGAFOS, processing stabilizer; BASF), and BYK-MAX LS 4125 (BYK, UV-stabilizer; BYK) as additives; and PV Fast Red D3G (Pigment Red 254; Clariant Plastics & Coatings GmbH) and PV Fast Blue BG-IN (Pigment Blue 15:3; Clariant Plastics & Coatings GmbH) as dyes. As fillers are normally added in high concentrations depending on the use case [33], we decided to add the fillers in higher concentrations of 40 *wt*%, 20 *wt*%, 10 *wt*%, 5 *wt*%, 2.5 *wt*%, and 1.25 *wt*%. This selection of concentrations allows a direct logarithmic dependence of the fluorescence lifetime on the concentration to be made during evaluation. Additives and dyes are usually added in low (< 1 *wt%*) concentrations. As mentioned in the data sheets of the selected additives and dyes, the typical used concentrations are 0.05–0.1 *wt%* (IRGANOX 1010), 0.05–0.15 *wt%* (IRGAFOS 126), 0.5–2 *wt%* (BYK-Max LS 4125), 0.01–0.1 *wt%* (PV Fast Red D3G), and 0.01–0.1 *wt%* (PV Fast Blue BG-IN). In the context of comparability and the logarithmic dependence of fluorescence lifetime and the different additive and dye concentrations, 8 *wt*%, 4 *wt*%, 2 *wt*%, 1 *wt*%, 0.5 *wt*%, and 0.25 *wt*% were used for the injection molding.

### Experimental setup

**Fig. 2:**
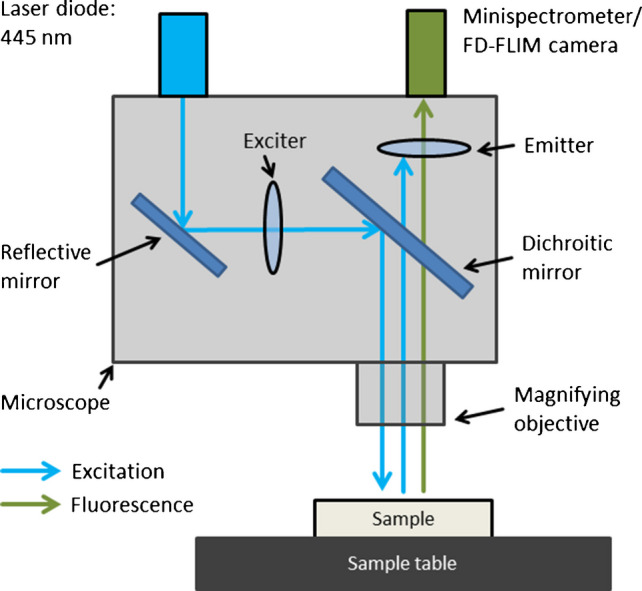
Experimental setup consisting of a laser diode including a 445-nm wavelength, either a Minispectrometer or an FD-FLIM camera, an optical band-pass filter (exciter), and an optical long-pass filter (emitter) assembled onto a microscope providing a ×20 magnification [28]

The experimental setup described by [28] is used for this experiment (see Fig. 2). It consists of a laser diode exhibiting an excitation wavelength of 445 nm of 500 mW, a Minispectrometer (Hamamatsu Photonics Deutschland GmbH), which is changeable with an FD-FLIM camera, a pco.flim (Excelitas PCO GmbH), a microscope EPAF150 (Cascade Microtech), and two optical filters: an exciter, which narrows the bandwidth of the exciting laser diode and an emitter, which blocks reflections and stray light, such that only the fluorescence is detected by the Minispectrometer/FD-FLIM camera. Furthermore, the microscope provides the possibility to choose between ×2, ×10, and ×20 magnification, whereby only the ×20 magnification was used during the investigations as it provides the highest accuracy.

### Measurement procedure and evaluation of fluorescence spectra

The measurement and evaluation procedure of fluorescence spectra are similar to those described in [28]. The Minispectrometer is assembled to the microscope to acquire the fluorescence spectra of the different plastics containing filler, additive, and dye concentrations. The exposure time of the Minispectrometer was set to 2 s, and the optical output power of the laser diode was adjusted to its maximum for every spectral measurement. This was done to make the fluorescence spectra comparable to each other. After completing the measurement settings, a dark measurement was performed, which is directly subtracted from the spectra of the plastic types, to eliminate environmental background noises. Five spectra of each plastic composition were acquired, averaged, and saved in a data table. A MATLAB script was programmed to evaluate the measurements, which automatically imports the data table and smooths and interpolates (interpolation step-size 0.01 nm) the spectral data. From the interpolated data calculating the maximum ${I}_{\mathrm{max}}$, the corresponding wavelength of the maximum ${\lambda }_{\mathrm{max}}$ and the Stokes shifts $\Delta \lambda$, defined with the subtraction of ${\lambda }_{\mathrm{Ex}}$ from ${\lambda }_{\mathrm{max}}$, is possible.

### Measurement procedure and evaluation of FD-FLIM

Also, the measurement procedure and evaluation of FD-FLIM measurements is similar to the procedure and evaluation introduced in [28] and was adapted for the present investigations. The FD-FLIM camera exchanges the Minispectrometer for the areal fluorescence lifetime measurements. Analogously to the spectral measurements, the optical output power of the laser diode is set to a maximum. The software NIS Elements from NIKON is used to set the required measurement parameters like exposure time and modulation frequency (30 MHz) as well as for the acquisition. A reference slide from Starna Scientific $({\tau }_{\mathrm{ref}}=3.75 ns)$ is placed on the microscope stage to reference the measurement setup.

After the experimental setup is referenced, the 126 samples containing different additive, filler, or dye concentrations are measured ten times in ten different measurement positions to omit any inhomogeneity of filler, additive, or dye in the plastic composite that could occur during the IM. Additionally, the pure IM plastics and the pure additives, fillers, and dyes are measured ten times. One FD-FLIM measurement results in a TIF (tagged image file) stack containing five channels: fluorescence intensity, phase shift, modulation index, and phase-dependent and modulation-dependent fluorescence lifetime.

To evaluate the ten resulting TIF stacks (containing the five channels) per sample, they are imported into MATLAB. For the ten TIF stacks, a total histogram is created for each of the five channels. After those are created, a Gaussian analysis is performed on the histogram for each channel until an *R*^2^ of 0.95 is reached or a maximum of five Gaussian distributions are fitted into the histogram of the channel data. This analysis results in five maxima, mean values, and standard deviations of each sample’s channel. Although the Gaussian analysis is done for each channel, whereby the phase-dependent fluorescence lifetime channel is the most reliable one and is thus used for evaluation.

To determine the influence of the filler, additive, or dye concentration, a numerical and graphical evaluation is implemented. For the numerical evaluation, a fitting algorithm of the proposed model (Eq. (4)) was programmed and judged with an *R*^2^ test. The results are then displayed in the graphical evaluation, representing the dependency of the filler, additive, or dye concentration on the phase-dependent fluorescence lifetime.

## Results and discussion

### Fluorescence spectroscopy

**Fig. 3:**
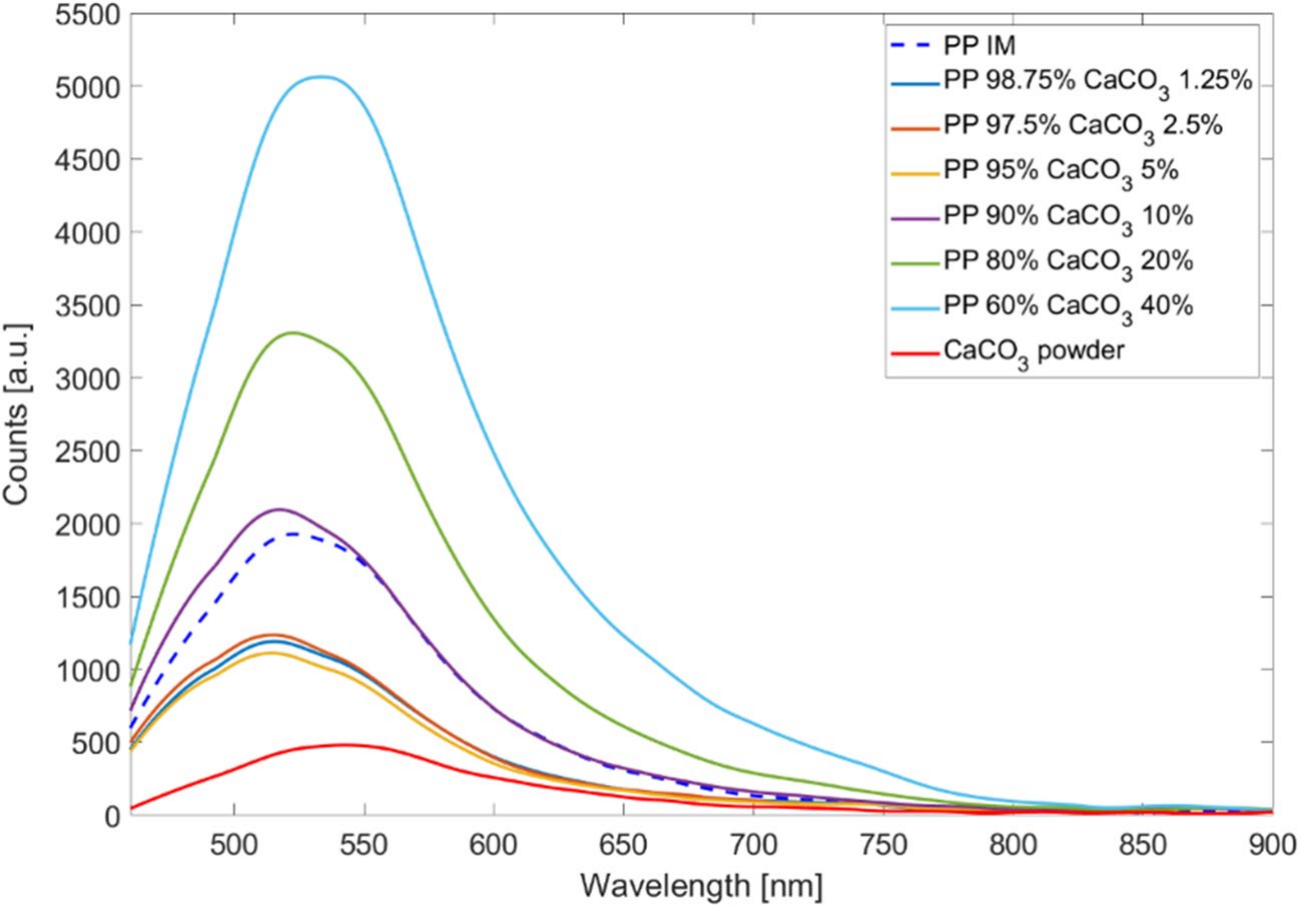
Fluorescence spectra of PP with different added concentrations of CaCO_3_, PP IM, and CaCO_3_ powder

Through this graphical and numerical evaluation, we were able to determine that the fluorescence intensity ${I}_{\mathrm{max}}$ of the PP/CaCO_3_ (Fig. 3), PS/CaCO_3_ (Figure S8), and LDPE/CaCO_3_ (Figure S15) composites increases linearly with increasing filler concentration (numerical evaluation Table 1, Table S1). Additionally, a decrease in the Stokes shift $\Delta \lambda$ for each plastic type mixed with CaCO_3_ was determined; i.e., the energy decrease due to vibrational relaxation is smaller. A similar behavior of the rising fluorescence intensity ${I}_{\mathrm{max}}$ with increasing filler concentration was obtainable for the composites of PP/talcum (Figure S2), PS/talcum (Figure S9), and LDPE/talcum (Figure S16). For talcum composites, the stokes shift $\Delta \lambda$ does not change (PP composite), decrease (PS composite), or increase (LDPE composite) (Table S1).

If the additive BYK is added to the three plastic types in different concentrations, the results of the spectral evaluation show an increase in fluorescence intensity the more BYK is added for the plastic types PS and LDPE (Figures S10 and S17). However, no dependency can be found when added to PP (Figure S3). Adding IRGAFOS to LDPE increases fluorescence intensity (Figure S18), but if added to PS and PP, no dependency on the fluorescence intensity of the concentration can be obtained (Figures S4 and S11). If IRGANOX is added to PP, PS, or LDPE, the fluorescence intensity is decreasing (Figure S5) with higher concentrations, stagnating (Figure S12) at all concentrations, or increasing (Figure S19) with higher concentrations of the additive, respectively. The entered values of the Stokes shift in Table S1 conclude that no dependency of additive concentration and Stokes shift could be determined.

The blue dye decreases fluorescence intensity independent of the plastic type (Figures S6, S14, S20, and Table S1). The concentration of the blue dye shows no dependency on the Stokes shift (Table S1). If the red dye is added to PP or LDPE, fluorescence intensity increases (Figures S7 and S15); if it is added to PS, a decrease in fluorescence intensity is obtained (Figure S21). Additionally, the red dye nearly completely quenches the fluorescence of the plastic types PP and LDPE. The Stokes shift decreases independently of the plastic type with lower red dye concentration (Table S1).

In conclusion, the results show that the filler, additive, and dye concentrations impact the spectral fluorescence response. However, only fillers show consistent dependencies of the fluorescence intensities and concentrations. For additives and dyes, no dependencies were obtainable. Additionally, determining a dependency of the compositions’ fluorescence from the pure materials’ (IM pure plastic types) fluorescence spectra was not possible for each material combination. Although Wohlschläger et al. [29] have shown that 445 nm is the optimal excitation wavelength with the currently used experimental setup to distinguish between pure plastic types, further excitation wavelengths should be investigated, e.g., 405 nm as Ornik et al. [34] showed the differentiability of different plastic types employing this excitation wavelength or excitation ranging from 285 to 320 nm as those are the optimal excitation wavelengths to differentiate between different plastic types according to Brackmann et al. [35].

**Table 1:** Results from analyzing the measured fluorescence spectra and calculating the ${I}_{\mathrm{max}}$, ${\lambda }_{\mathrm{max}}$, and $\Delta \lambda$ values

Compound	Concentration *wt*%	${{{I}}}_{\mathrm{max}}$	${{{\lambda}}}_{\mathrm{max}}$	${{\Delta}}{{\lambda}}$
PP	Pure IM	1927	523	78
PP/CaCO_3_	60.00/40.00	5061	534	89
PP/CaCO_3_	80.00/20.00	3305	523	78
PP/CaCO_3_	90.00/10.00	2095	518	73
PP/CaCO_3_	95.00/5.00	1112	515	70
PP/CaCO_3_	97.50/2.50	1236	515	70
PP/CaCO_3_	98.75/1.25	1191	515	70
CaCO_3_	Powder	480	543	98

### Results and discussion

#### FD-FLIM measurements

The determined phase-dependent fluorescence lifetimes ${\tau }_{\mathrm{PH}} \left[ns\right]$ of the various composites, the pure IM plastics, and the fillers, additives, and dyes in their original form are entered in Tables 2 and S2. The calculated ${\tau }_{\mathrm{PH}} \left[ns\right]$ of all filler composites (PP/CaCO_3_, PS/CaCO_3_, LDPE/CaCO_3_, PP/talcum, PS/talcum, and LDPE/talcum) show a slower decaying of the fluorescence by increasing filler concentrations (Example images of the phase-dependent fluorescence lifetime of measurement of PP/CaCO_3_ at concentration 98.75/1.25 are added to Figure S1). Contrarily, PP/BYK, PS/BYK, LDPE/BYK, PP/IRGAFOS, PS/IRGAFOS, LDPE/IRGAFOS, PP/IRGANOX, and LDPE/IRGANOX composites show a similar increase in fluorescence lifetimes when more additives are added. A different behavior is obtained from the PS/IRGANOX composite, showing stagnation of fluorescence lifetimes in one standard deviation. The fluorescence lifetimes of the dye/plastic compositions decay faster the more dye is added, except for RED/LDPE, where the fluorescence lifetime stagnates at 0.3 ns.

In principle, a dependency of the phase-dependent fluorescence lifetime from the filler, additive, and dye concentrations can be seen by Gaussian evaluation of the FD-FLIM images. However, the measured fluorescence lifetimes of the pure IM plastics and the fillers, additives, and dyes in their original form do not provide any information on how this dependence of concentration and fluorescence lifetime develops, as these are either larger or smaller than the measured fluorescence decay times of composites. Additionally, if the determined phase-dependent fluorescence lifetimes of the IM pure PP (1.88 ± 0.06) and PS (2.01 ± 0.06) are compared to the reported phase-dependent fluorescence lifetimes of PP (1.35 ± 0.17) and PS (1.50 ± 0.12) granulates from [29], an increase of 0.5 ns is caused by the injection molding process.

**Table 2:** Results from Gaussian analysis of the phase-dependent fluorescence lifetime ${\tau }_{\mathrm{PH}} [ns]$ from ten FD-FLIM images per sample

Compound	Concentration *wt%*	${{{\tau}}}_{\mathrm{PH}}\boldsymbol{ }[{{n}}{{s}}]$
PP	IM	1.88 ± 0.06
PP/CaCO_3_	60.00/40.00	2.36 ± 0.05
PP/CaCO_3_	80.00/20.00	2.44 ± 0.07
PP/CaCO_3_	90.00/10.00	2.34 ± 0.07
PP/CaCO_3_	95.00/5.00	2.28 ± 0.08
PP/CaCO_3_	97.50/2.50	2.24 ± 0.07
PP/CaCO_3_	98.75/1.25	2.08 ± 0.07
CaCO_3_	Powder	1.95 ± 0.23

#### Modelling of the dependency

**Fig. 4:**
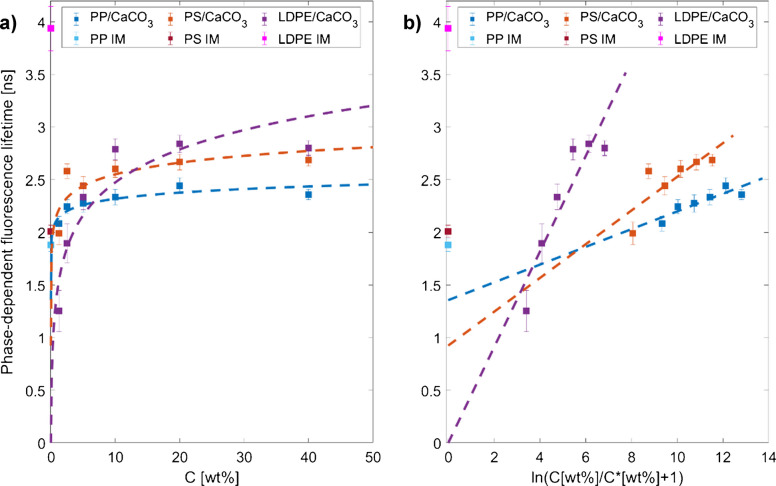
**a** Dashed lines show the logarithmic fit from Eq. (4) onto the measured fluorescence lifetimes of the composites PP/CaCO_3_, PS/CaCO_3_, and LDPE/CaCO_3_. The fluorescence lifetimes of the pure IM plastics are displayed at 0% concentration. **b** The fits in a logarithmic × scaling, whereby it can be seen that the fluorescence lifetimes of the pure IM materials cannot be related to the behavior of the fluorescence lifetimes of the composites. The squares represent the mean value of the ten FD-FLIM measurements per sample, while the error bars represent the standard deviation

We applied Eq. (4) from Langhals and Schlücker to our measured phase-dependent fluorescence lifetimes of the different composites [32]. The fluorescence lifetime of the pure IM plastic types was neglected during the modeling, as we could not see any dependencies of these and the fluorescence lifetimes of the composites. Figure 4a shows the logarithmic fit, and Fig. 4b shows the linear correlation of our determined fluorescence lifetimes and the concentration of filler for the composites PP/CaCO_3_, PS/CaCO_3_, and LDPE/CaCO_3_. From graphically evaluating the diagrams in Fig. 4a and b, we derived that the model from Eq. (4) represents the dependency of the fluorescence lifetimes of PP, PS, and LDPE on the CaCO_3_ concentration with a high correlation. However, Fig. 4 also shows that identifying the plastic types becomes difficult because the increase in filler content superimposes the fluorescence lifetimes of the different plastic types. The calculated *R*^2^ values in Table 3 confirm that the model fits the fluorescence lifetime/concentration dependency. However, only an accuracy of 63% is obtained for PS/CaCO_3_, which may have been caused by errors when weighing the amount of filler for IM. However, the correlation of fluorescence lifetime and concentration can be precisely described with an accuracy of 79–85% for PP/CaCO_3_ and LDPE/CaCO_3_, respectively.

The results of determining the dependency for the other composites are shown in Figures S22–S27. These graphical representations also show that identifying the plastic types by measuring the fluorescence lifetime via FD-FLIM is difficult due to the superimposition of the fluorescence lifetimes at increasing additive, filler, and dye concentrations. Additionally, the determined parameters of the fitted model from Eq. (4) and the corresponding *R*^2^ values for each fit are entered in Table 3. We derived that the presented model has a high correlation (80 to 100%) for 76% of composites. No correlation was found for PS/IRGANOX and LDPE/Red compositions, achieving only an *R*^2^ value of 46% and 31%, respectively. The low correlations of PS/IRGANOX and PP/Red of about 65–66% could be caused by errors in the weighing for IM.

In conclusion, we found that most composites can be represented by the model from Eq. (4), which is in accordance with the model from Langhals and Schlücker [32]. Thus, as our results demonstrate, the concentration dependency of the fluorescence lifetime for a mixture of materials should be questioned in its original form described by the Strickler-Berg equation [25], because of the findings of Langhals and Schlücker [32] and our determined results.

**Table 3:** Determined parameters for the fitted model from Eq. (4) and *R*^2^ value

Composite	*a* [ns]	C* [%]	${\tau }_{0}$ [*ns*]	*R*^2^
PP/CaCO_3_	0.08	2.01 × 10^–4^	1.41	0.79
PS/CaCO_3_	0.16	3.78 × 10^–4^	0.92	0.63
LDPE/CaCO_3_	0.45	0.04	0.24 × 10^–11^	0.85
PP/talcum	0.08	2.70 × 10^–4^	1.22	0.89
PS/talcum	0.07	0.01	1.88	0.85
LDPE/talcum	0.19	4.90 × 10^–4^	0.30	0.82
PP/BYK	0.16	0.89	1.97	0.98
PS/BYK	0.42	0.53	2.02	0.96
LDPE/BYK	0.76	0.43	0.26	0.99
PP/IRGAFOS	3.68	35.08	2.00	0.99
PS/IRGAFOS	0.11	2.25 × 10^–4^	1.76	0.66
LDPE/IRGAFOS	0.35	0.01	6.24 × 10^–4^	0.99
PP/IRGANOX	0.67	1.97	10.06	0.99
PS/IRGANOX	−0.10	2.92	0.64 × 10^–4^	0.46
LDPE/IRGANOX	0.61	0.17	1.98	1.00
PP/blue	−0.33	2.64 × 10^–4^	3.40	0.92
PS/blue	−0.13	10.69 × 10^–4^	2.12	0.98
LDPE/blue	−0.69	2.55	1.16	0.99
PP/red	−0.09	1.84 × 10^–4^	1.16	0.65
PS/red	−1.14	0.29	4.92	1.00
LDPE/red	−0.01	0.05	0.33	0.31

## Conclusion

Today, there is a great need for new, reliable, and fast analytical methods to directly identify MP particle properties such as plastic type, shape, and size in environmental samples to assess the contamination level and thus ensure a comprehensive ecological risk assessment.

Measuring the fluorescence lifetime in TD or FD could be a novel analytical method to assess the essential parameters of the microplastic in extracted samples with fewer purifications. Previous studies showed that identifying plastic type [26, 27], differentiating those from natural materials [24, 28, 30], and obtaining their shape and size at least down to a size of 70 µm on soil [28] are possible using TD or FD-FLIM. However, the impact of filler, additive, and dye concentrations on the identifiability of plastic types has yet to be researched. Therefore, we investigated (I) whether the dependence of the fluorescence lifetime/concentration can be heuristically modeled and (II) if an identification of the plastic type is still possible although additive, filler, and dye concentrations are added via FD-FLIM.

In this context, we were able to answer our research questions: (I) heuristically modeling the dependency of the fluorescence lifetime of filler, additive, and dye concentrations by applying the proposed model from Langhals and Schlücker is possible with a high correlation; however, (II) identifying the plastic types is hardly possible due to the superimposition of the fluorescence lifetimes at an increasing filler, additive, and dye concentrations. Hence, directly identifying plastic types via FD-FLIM in an environmental matrix is only possible if further investigations on the dependency of the fluorescence lifetime from filler, additive, and dye concentrations are performed, as only three plastic types in combination with two fillers, three additives, and two dyes have been investigated. Therefore, future investigations should include more plastic types composed of different fillers, additives, and dyes. Additionally, as we measured the fluorescence lifetimes of the whole fluorescence spectra, investigating different emission states may be a solution to the overlaying fluorescence lifetimes. Furthermore, an extended study where other wavelengths, e.g., those tested by [34, 35], are used to determine the impact of additive, filler, and dye concentrations in plastics on the fluorescence spectra, would be desirable. Moreover, other fluorescence lifetime measurement methods (e.g., TCSPC, TD-FLIM) should be used to validate the presented results. Executing these investigations may make FD-FLIM a promising method for directly identifying plastic types in environmental matrices. In addition, machine learning should be introduced based on the already gathered results as it was already reported as promising for plastic type identification using photoluminescence spectroscopy [36] and FD-FLIM images [30].

Although a direct identification of plastic types in environmental matrices seems hardly possible via FD-FLIM, other fields to apply FD-FLIM arise. As our results show, FD-FLIM is a highly sensitive measurement method to determine the filler, additive, and dye concentrations, which could be advantageous in plastics manufacturing and plastics recycling during manufacturing. In foresight, two possible applications may be including FD-FLIM to monitor the homogeneity of filler, additive, or dye concentrations during plastics manufacturing. The second application could be to identify plastic types with specific colorant concentrations from faulty productions to return them directly to the production process as recyclate.

## Supplementary Information

Below is the link to the electronic supplementary material.

Supplementary file1 (PDF 3.79 MB)

## Data Availability

The data will be available from the authors upon request.
