# Supplementary material for: Determining the influence of variable additive, filler, and dye concentrations in plastics on their fluorescence behavior via spectrometry and FD-FLIM
Source: Anal Bioanal Chem. 2026 Feb 5;418(7):1961–71. doi: 10.1007/s00216-026-06361-0 (PMC12999643; doi:10.1007/s00216-026-06361-0)
Supplement: Supplementary file 1 — Supplementary file1 (PDF 3.79 MB) [file 216_2026_6361_MOESM1_ESM.pdf]

Determining the influence of variable additive, filler, and dye concentrations in plastics on their fluorescence behavior via spectrometry and FD-FLIM

Author list

Maximilian Wohlschläger[1], Markus Bonauer[2], Manuela List[2], Martin Versen[1], Martin G.J. Löder[3], Christian Laforsch[3]

[1] Faculty of Engineering Sciences, Rosenheim Technical University of Applied Sciences, Hochschulstraße 1, 83024 Rosenheim, Germany.

[2] Faculty of Chemical Technology and Economics, Rosenheim Technical University of Applied Sciences (Campus Burghausen), Robert-Koch-Straße 28, 84489 Burghausen, Germany.

[3] Animal Ecology I and BayCEER, University Bayreuth, Universitätsstraße 30, 95440 Bayreuth, Germany.

Corresponding author: maximilian.wohlschläger@th-rosenheim.de

The supporting Information consists of 27 Figures: Figure S1 shows an example image of the phase-dependent fluorescence lifetime of PP/$CaCO_3$ at various concentrations. Figure S2 to Figure S21 shows the spectral fluorescence behavior of the various manufactured plastic compositions. Figure S22 to Figure S27 shows the fluorescence lifetime/concentration dependency, including the logarithmically fitted model. Additionally, two Tables are included in the SI: Table S1 providing the calculated spectral parameters (I_{max} [a.u.]; λ_{max} [nm]; $\Delta\lambda$ [nm]) from the analysis of the fluorescence spectra of the various manufactured plastic compositions. Furthermore, the calculated phase-dependent fluorescence lifetimes of the various composites are entered in Tables S2. In total, there are twenty two pages.

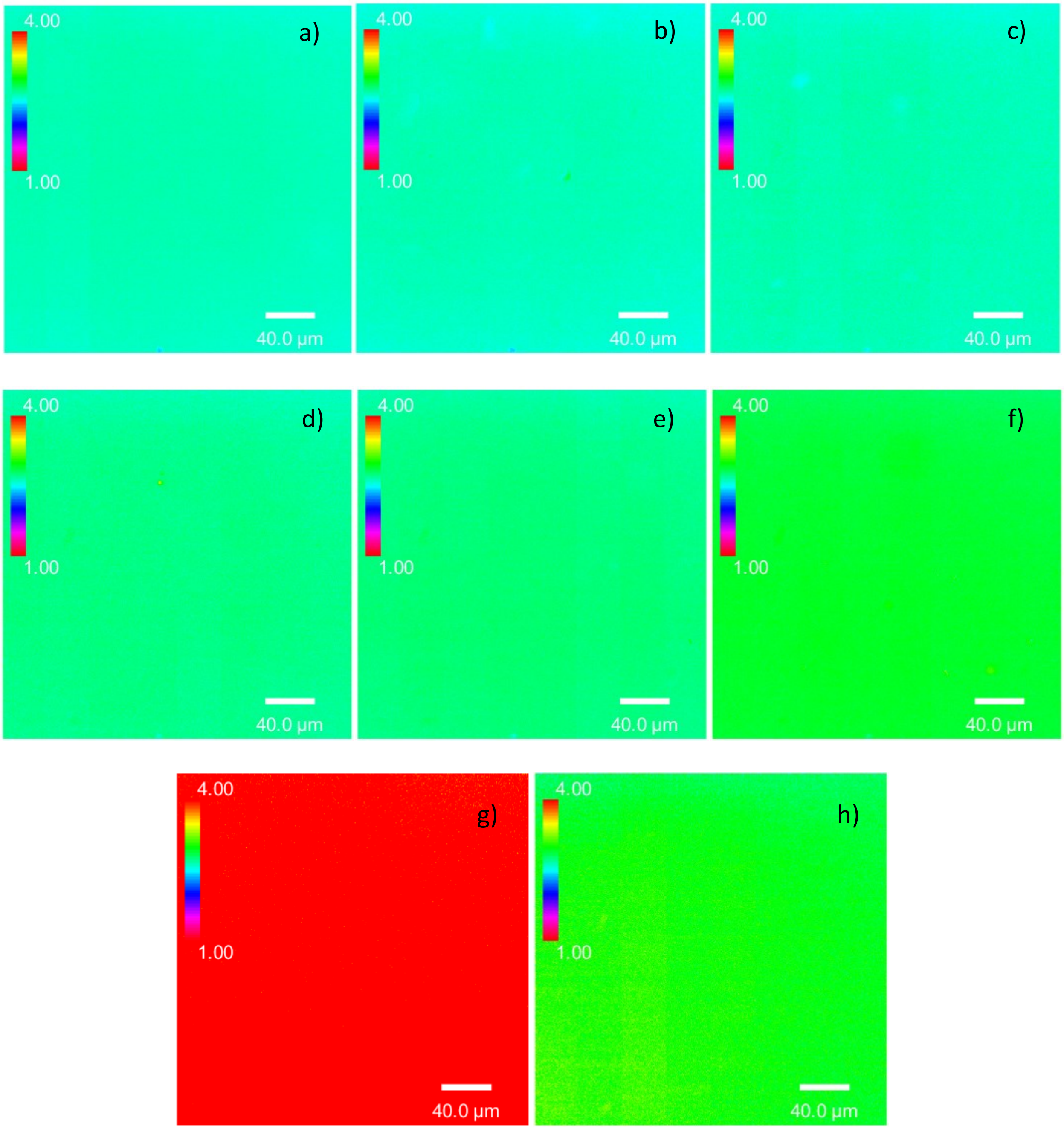

Figure S1: Example images of the phase-dependent fluorescence lifetime of PP/CaCO3 at concentration a) 60/40, b) 80/20, c) 90/10, d) 95/5, e) 97.5/2.5, and f) 98.75/1.25 as well as the pure materials g) PP and h) CaCO3

PP:

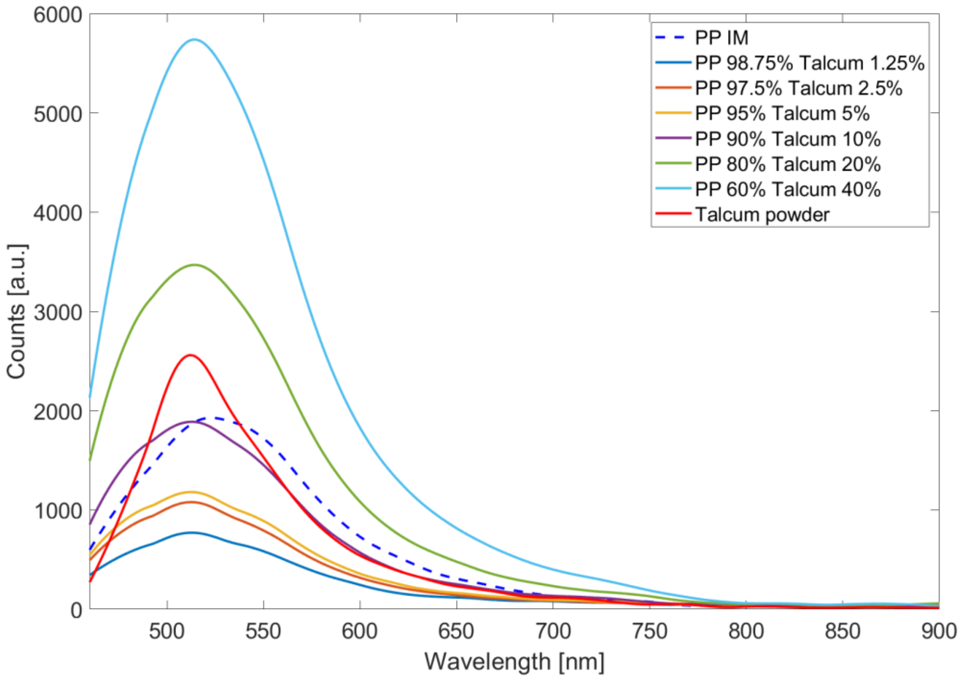

Figure S2. Fluorescence spectra of PP with different added concentrations of talcum, PP injection molded and talcum powder.

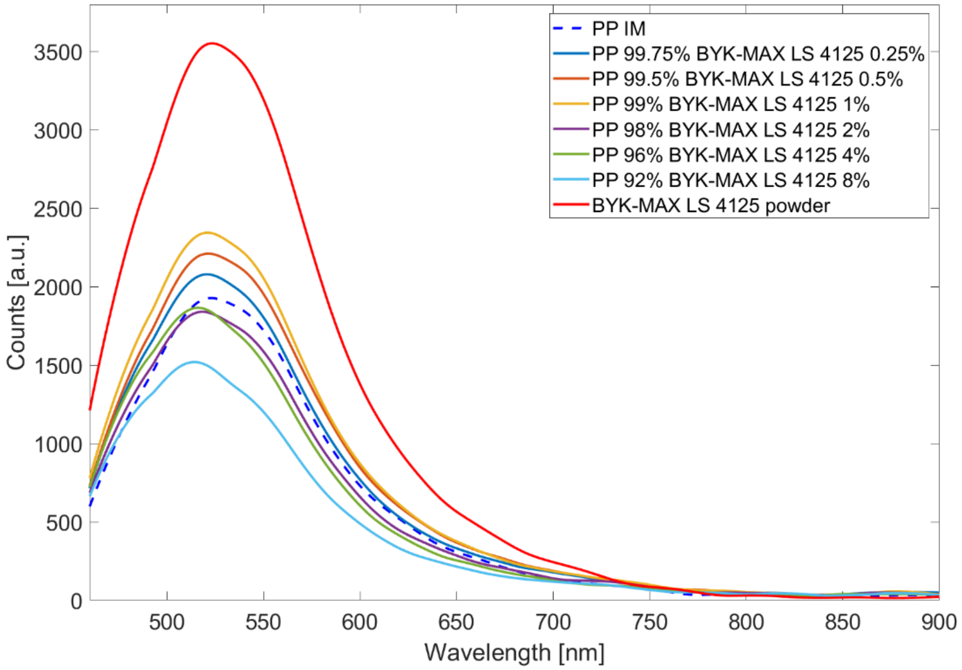

Figure S3. Fluorescence spectra of PP with different added concentrations of BYK-MAX LS 4125, PP injection molded and BYK-MAX LS 4125 granules.

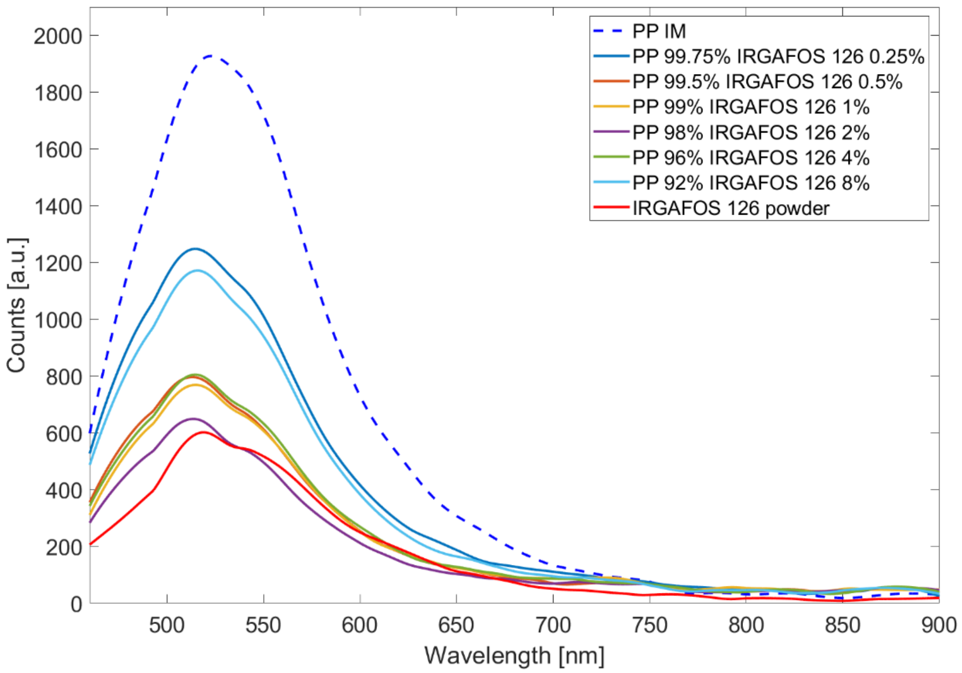

Figure S4. Fluorescence spectra of PP with different added concentrations of IRGAFOS 126, PP injection molded and IRGAFOS 126 powder.

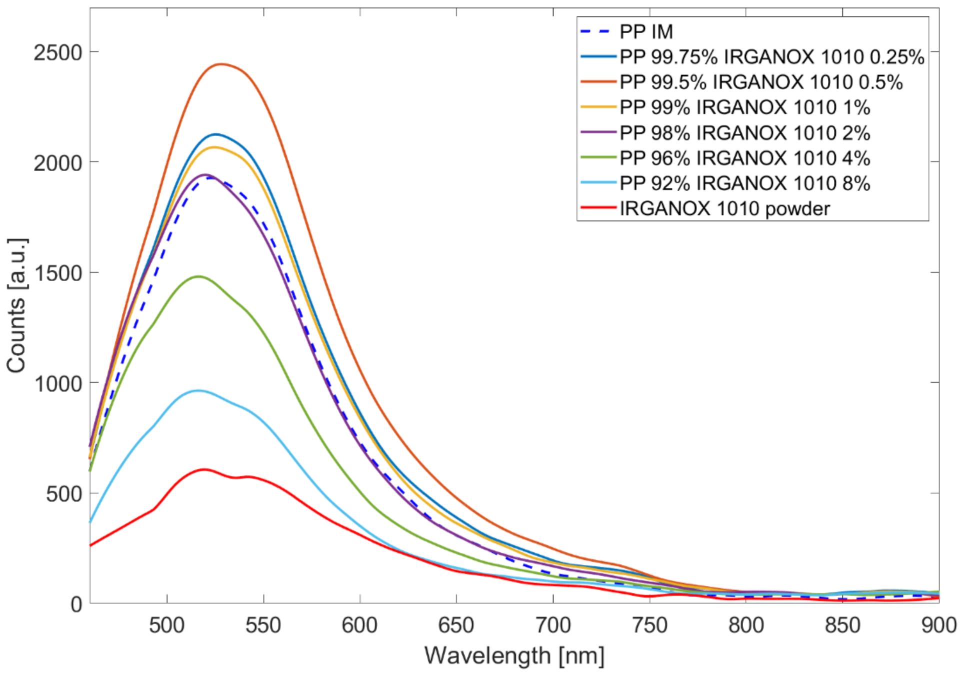

Figure S5. Fluorescence spectra of PP with different added concentrations of IRGANOX 1010, PP injection molded and IRGANOX 1010 powder.

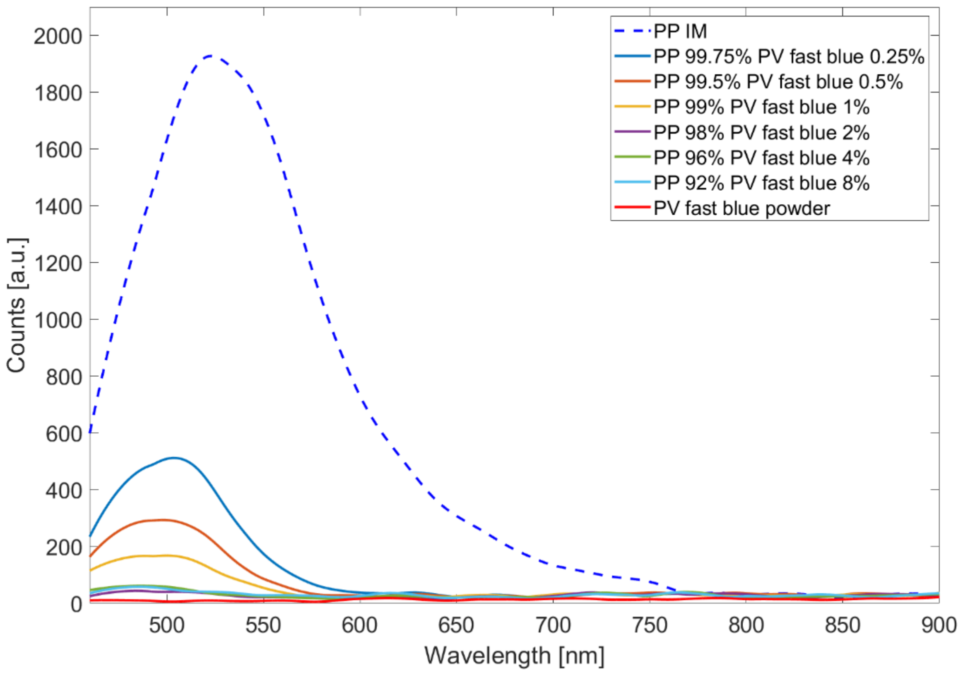

Figure S6.Fluorescence spectra of PP with different added concentrations of PV fast blue, PP injection molded and PV fast blue powder.

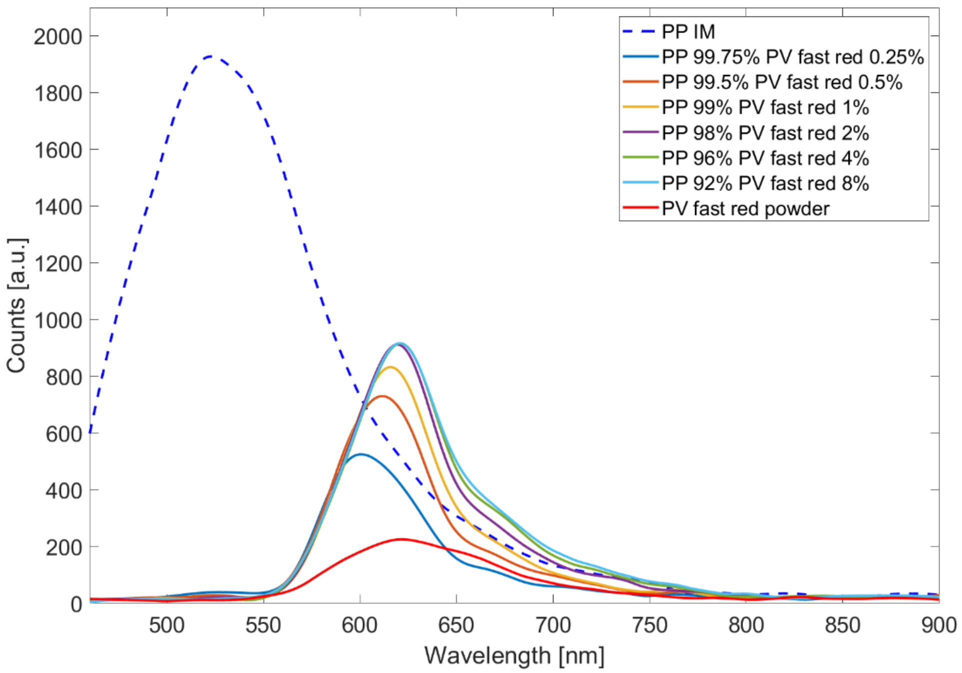

Figure S7.Fluorescence spectra of PP with different added concentrations of PV fast red, PP injection molded and PV fast red powder.

PS:

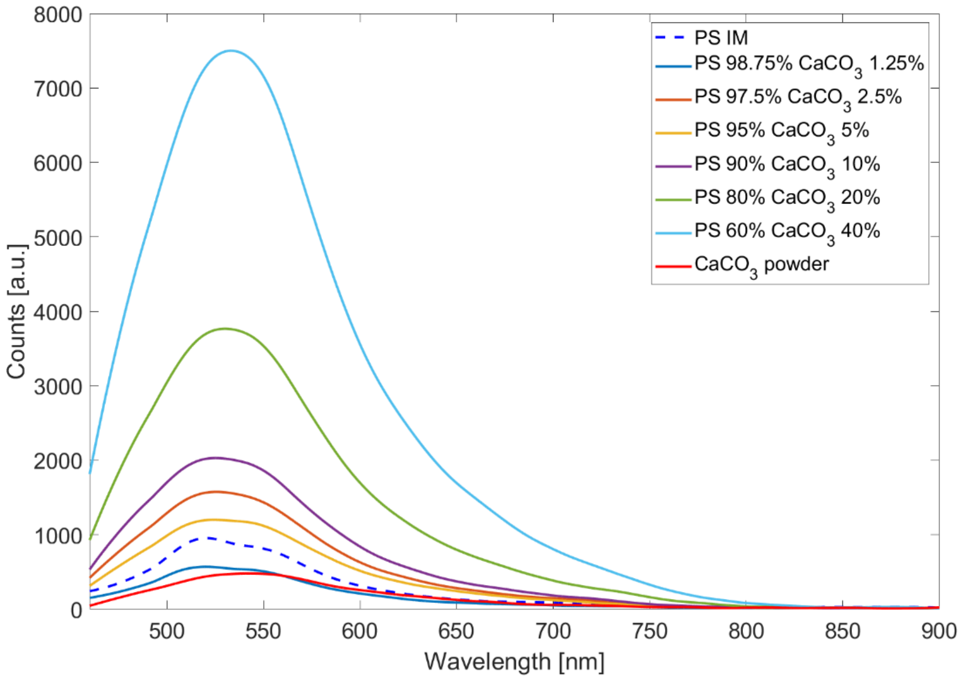

Figure S8. Fluorescence spectra of PS with different added concentrations of $CaCO_3$, PS injection molded and $CaCO_3$ powder.

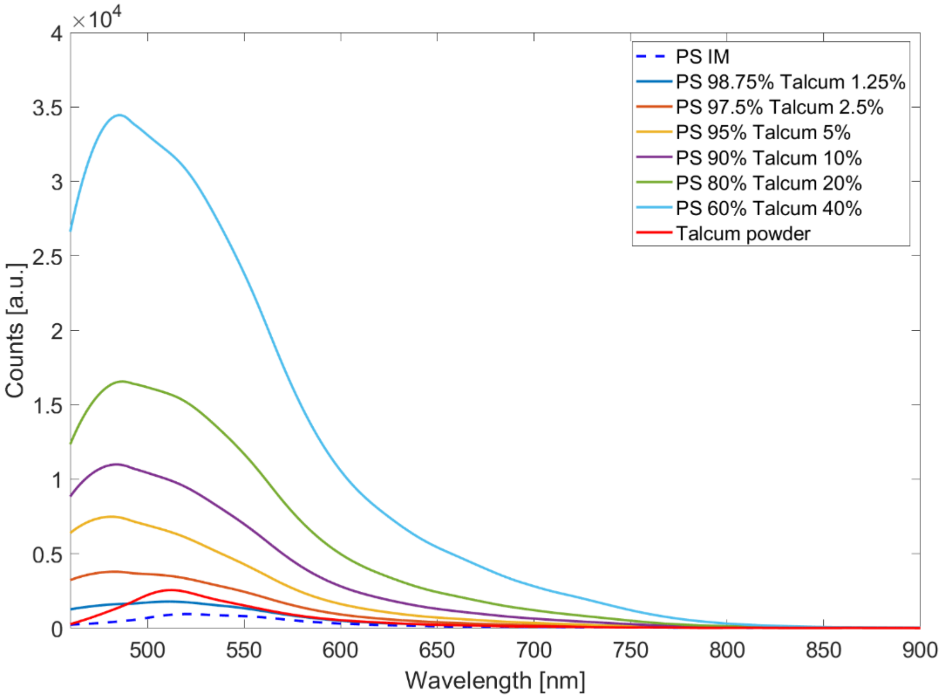

Figure S9. Fluorescence spectra of PS with different added concentrations of talcum, PS injection molded and talcum powder.

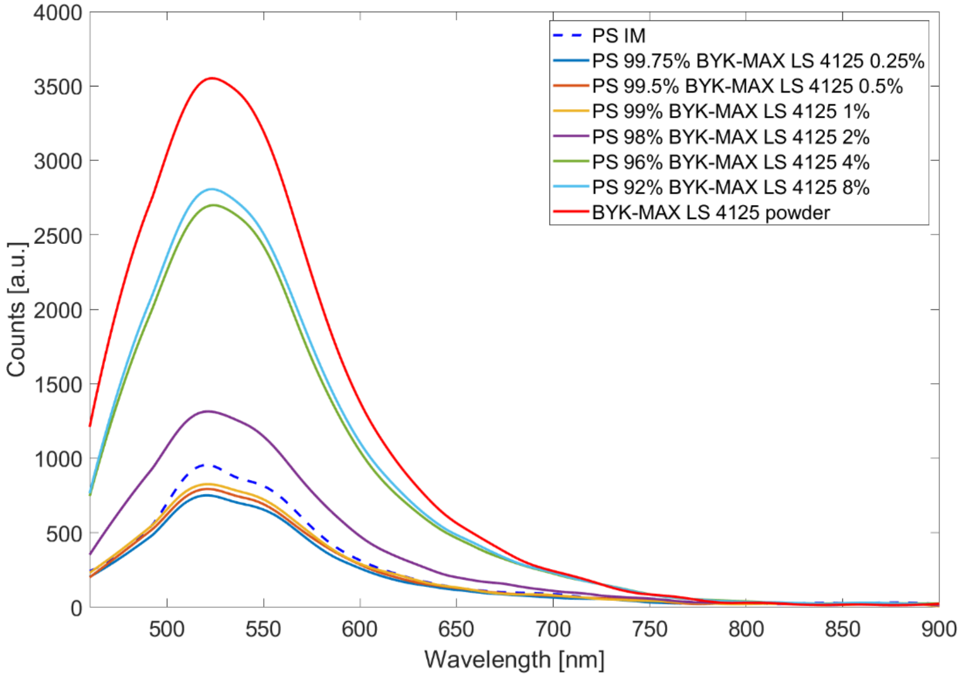

Figure S10. Fluorescence spectra of PS with different added concentrations of BYK-MAX LS 4125, PS injection molded and BYK-MAX LS 4125 granules.

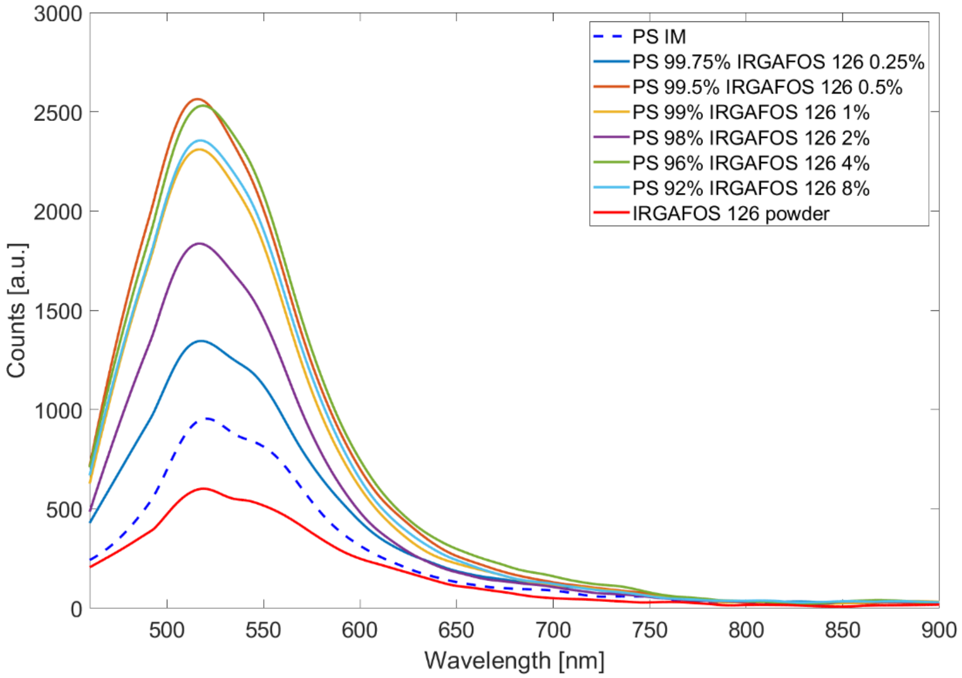

Figure S11. Fluorescence spectra of PS with different added concentrations of IRGAFOS 126, PS injection molded and IRGAFOS 126 powder.

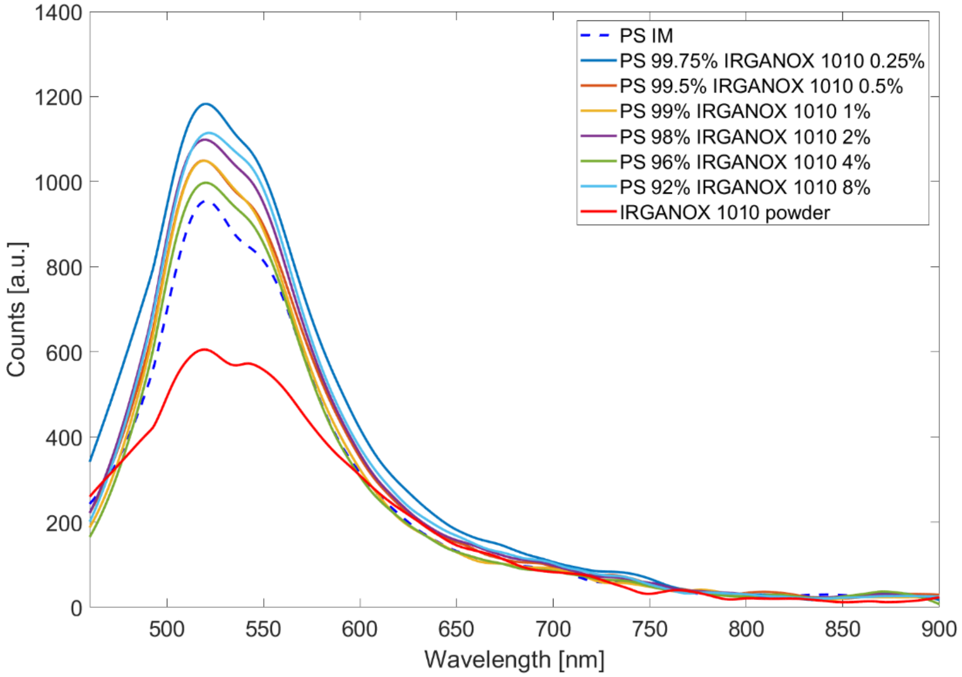

Figure S12. Fluorescence spectra of PS with different added concentrations of IRGANOX 1010, PS injection molded and IRGANOX 1010 powder.

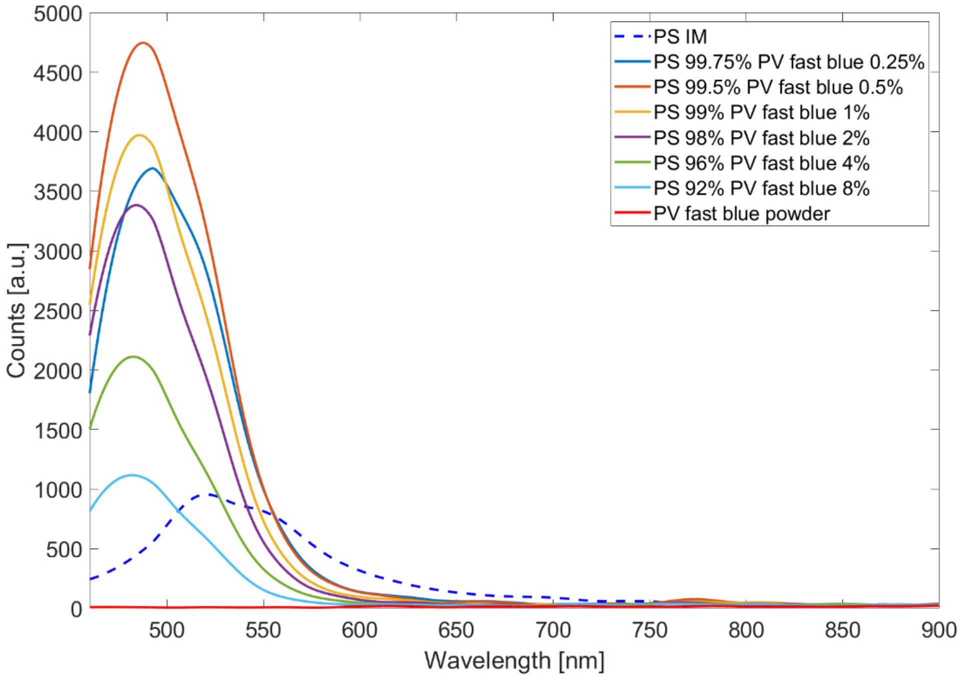

Figure S13. Fluorescence spectra of PS with different added concentrations of PV fast blue, PS injection molded and PV fast blue powder.

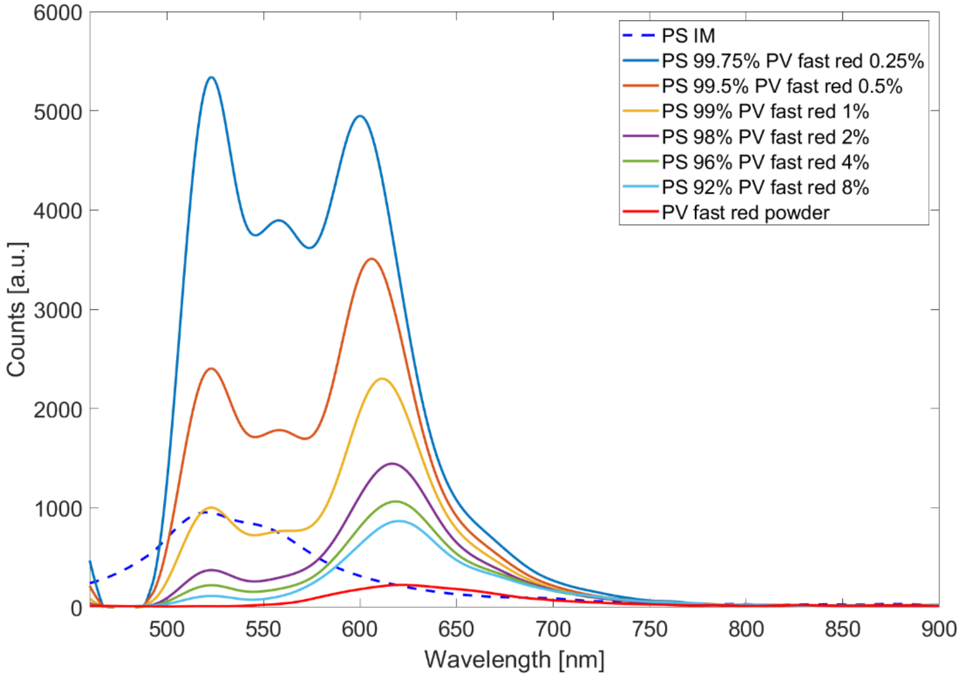

Figure S14. Fluorescence spectra of PS with different added concentrations of PV fast red, PS injection molded and PV fast red powder.

LDPE:

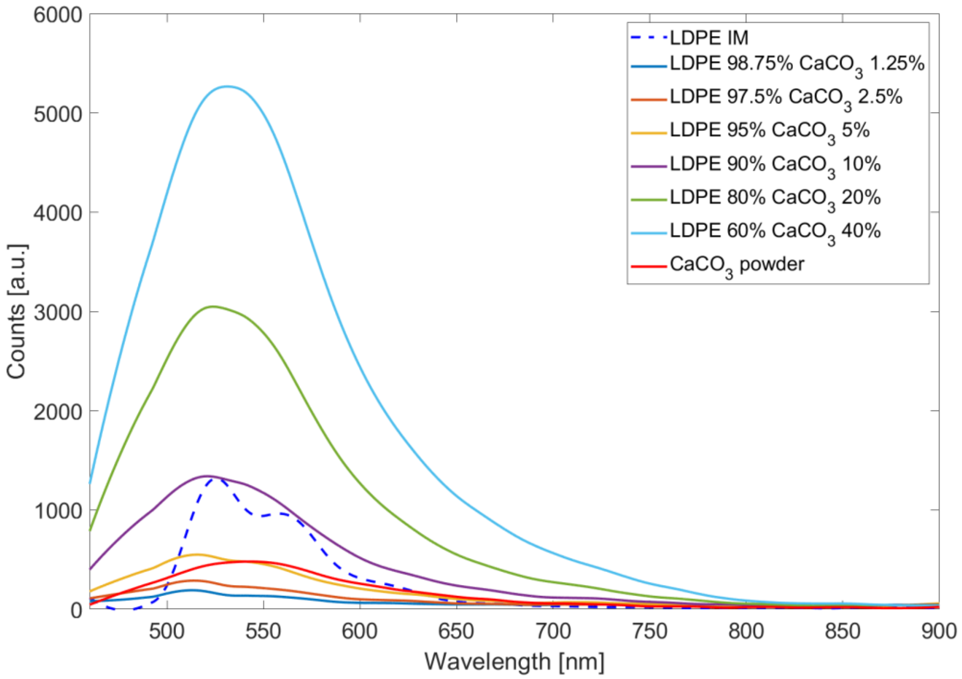

Figure S15. Fluorescence spectra of LDPE with different added concentrations of $CaCO_3$, LDPE injection molded and $CaCO_3$ powder.

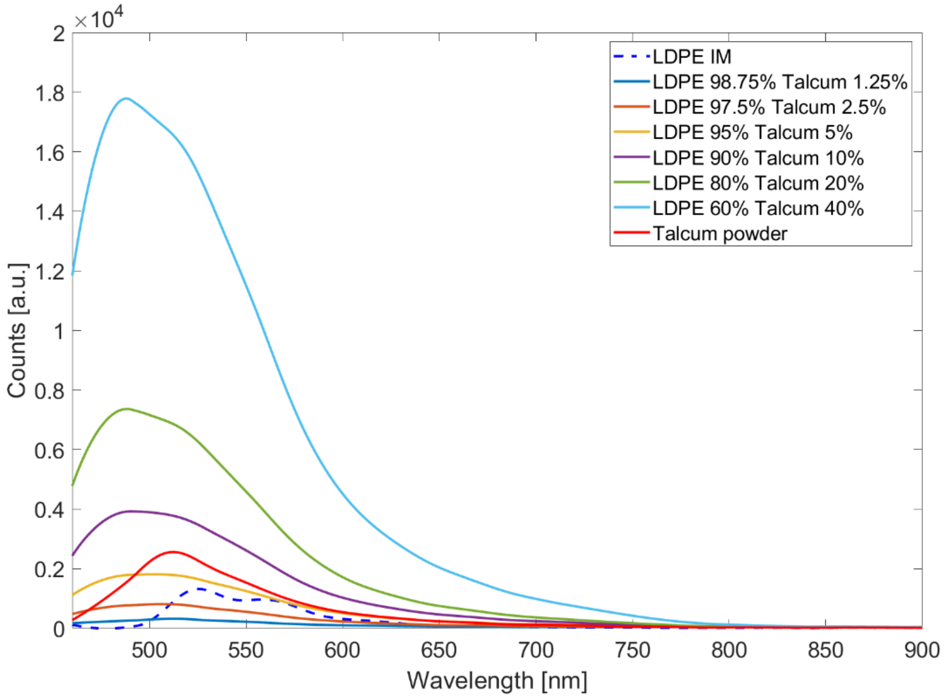

Figure S16. Fluorescence spectra of LDPE with different added concentrations of talcum, LDPE injection molded and talcum powder.

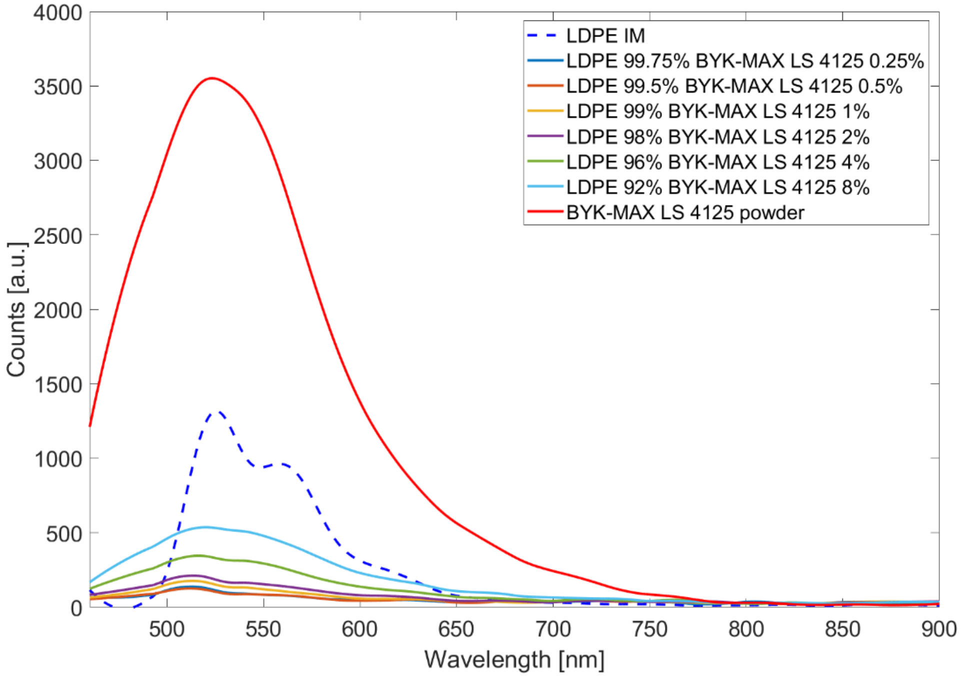

Figure S17. Fluorescence spectra of LDPE with different added concentrations of BYK-MAX LS 4125, LDPE injection molded and BYK-MAX LS 4125 granules.

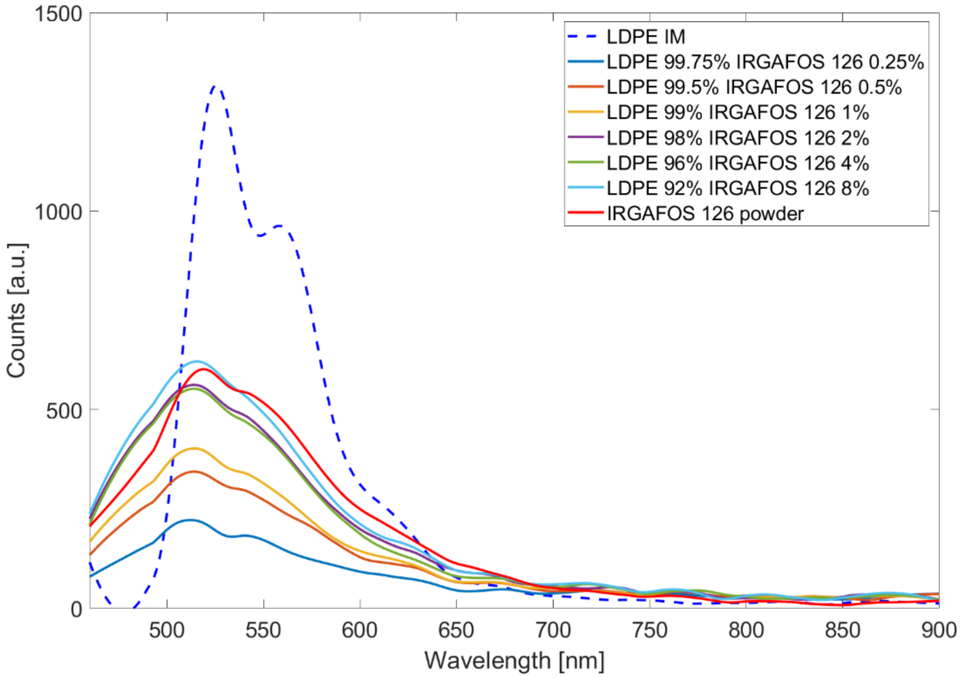

Figure S18. Fluorescence spectra of LDPE with different added concentrations of IRGAFOS 126, LDPE injection molded and IRGAFOS 126 powder.

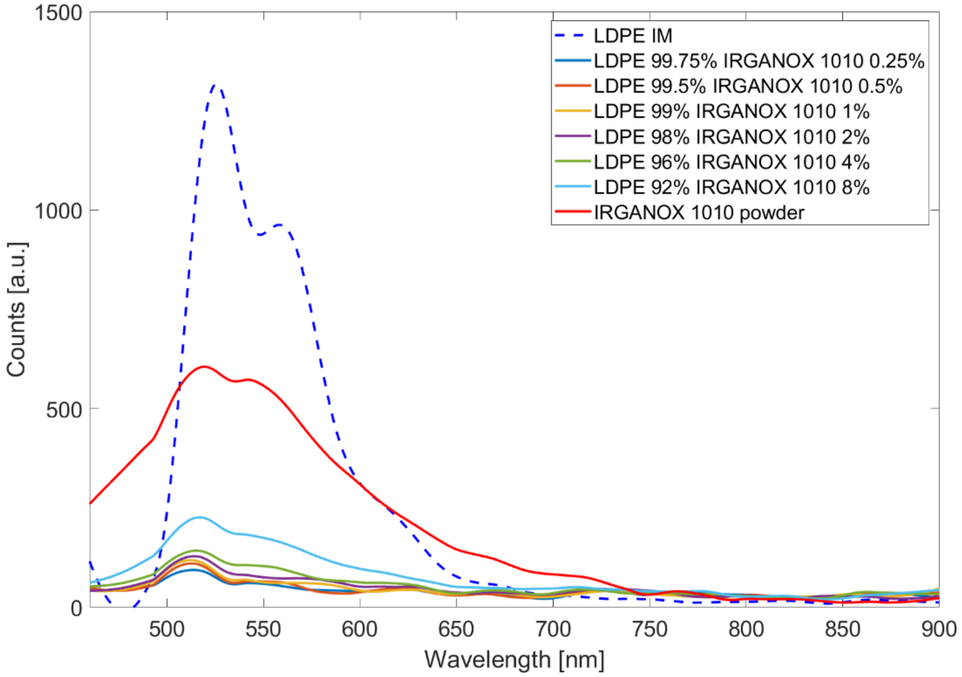

Figure S19. Fluorescence spectra of LDPE with different added concentrations of IRGANOX 1010, LDPE injection molded and IRGANOX 1010 powder.

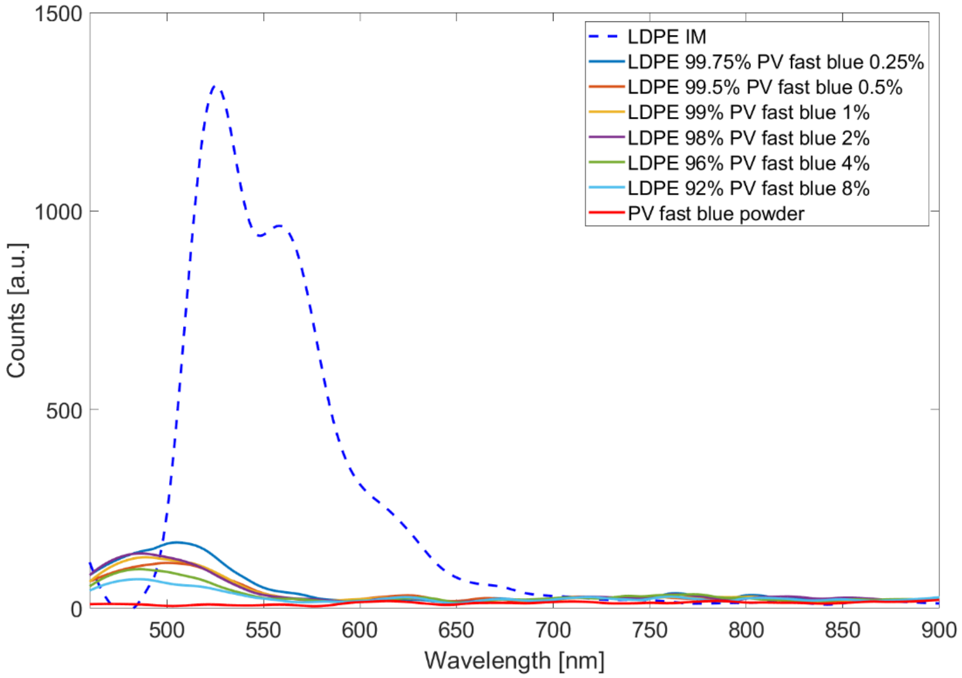

Figure S20. Fluorescence spectra of LDPE with different added concentrations of PV fast blue, LDPE injection molded and PV fast blue powder.

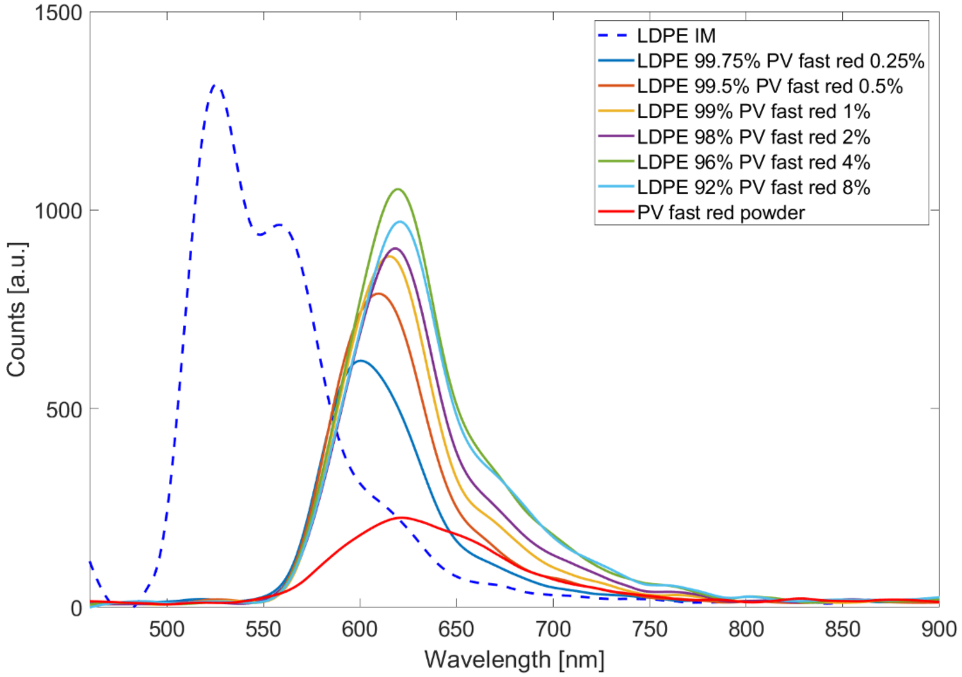

Figure S21. Fluorescence spectra of LDPE with different added concentrations of PV fast red, LDPE injection molded and PV fast red powder.

Table S1. Results from analyzing the measured fluorescence spectra and calculating the values for I_{max}, λ_{max} and $\Delta\lambda$.

**Compound**	**Concentration wt%**	I_{max}	λ_{max}	$\Delta\lambda$
**PP**	IM	1927	523	78
**PP/Talcum**	60.00/40.00	5738	514	69
**PP/Talcum**	80.00/20.00	3468	514	69
**PP/Talcum**	90.00/10.00	1888	513	68
**PP/Talcum**	95.00/5.00	1180	513	68
**PP/Talcum**	97.50/2.50	1079	513	68
**PP/Talcum**	98.75/1.25	771	513	68
**PP/BYK**	92.00/8.00	1519	514	69
**PP/BYK**	94.00/4.00	1866	516	71
**PP/BYK**	98.00/2.00	1840	518	73
**PP/BYK**	99.00/1.00	2344	521	76
**PP/BYK**	99.50/0.50	2211	521	76
**PP/BYK**	99.75/0.25	2079	521	76
**PP/IRGAFOS**	92.00/8.00	1172	516	71
**PP/IRGAFOS**	94.00/4.00	805	515	70
**PP/IRGAFOS**	98.00/2.00	649	514	69

**PP/IRGAFOS**	99.00/1.00	769	515	70
**PP/IRGAFOS**	99.50/0.50	796	513	68
**PP/IRGAFOS**	99.75/0.25	1248	515	70
**PP/IRGANOX**	92.00/8.00	964	516	71
**PP/IRGANOX**	94.00/4.00	1481	516	71
**PP/IRGANOX**	98.00/2.00	1941	520	75
**PP/IRGANOX**	99.00/1.00	2067	525	80
**PP/IRGANOX**	99.50/0.50	2443	528	83
**PP/IRGANOX**	99.75/0.25	2125	525	80
**PP/PV blue**	92.00/8.00	58	486	41
**PP/PV blue**	94.00/4.00	61	486	41
**PP/PV blue**	98.00/2.00	44	484	39
**PP/PV blue**	99.00/1.00	168	500	55
**PP/PV blue**	99.50/0.50	293	498	53
**PP/PV blue**	99.75/0.25	512	504	59
**PP/PV red**	92.00/8.00	916	621	176
**PP/PV red**	94.00/4.00	916	621	176
**PP/PV red**	98.00/2.00	912	619	174
**PP/PV red**	99.00/1.00	832	616	171
**PP/PV red**	99.50/0.50	730	612	167
**PP/PV red**	99.75/0.25	525	601	156
**PS**	granulate	955	521	76
**PS/$CaCO_3$**	60.00/40.00	7500	533	88
**PS/$CaCO_3$**	80.00/20.00	3767	530	85
**PS/$CaCO_3$**	90.00/10.00	2031	525	80
**PS/$CaCO_3$**	95.00/5.00	1201	524	79
**PS/$CaCO_3$**	97.50/2.50	1576	526	81
**PS/$CaCO_3$**	98.75/1.25	571	520	75
**PS/Talcum**	60.00/40.00	34452	486	41
**PS/Talcum**	80.00/20.00	16568	487	42
**PS/Talcum**	90.00/10.00	10996	484	39
**PS/Talcum**	95.00/5.00	7489	481	36
**PS/Talcum**	97.50/2.50	3795	483	38
**PS/Talcum**	98.75/1.25	1791	511	66
**PS/BYK**	92.00/8.00	2807	523	78
**PS/BYK**	94.00/4.00	2699	524	79
**PS/BYK**	98.00/2.00	1314	521	76
**PS/BYK**	99.00/1.00	826	521	76
**PS/BYK**	99.50/0.50	794	521	76
**PS/BYK**	99.75/0.25	751	521	76
**PS/IRGAFOS**	92.00/8.00	2356	517	72
**PS/IRGAFOS**	94.00/4.00	2531	519	74
**PS/IRGAFOS**	98.00/2.00	1836	517	72
**PS/IRGAFOS**	99.00/1.00	2310	517	72
**PS/IRGAFOS**	99.50/0.50	2564	516	71
**PS/IRGAFOS**	99.75/0.25	1346	518	73
**PS/IRGANOX**	92.00/8.00	1115	522	77
**PS/IRGANOX**	94.00/4.00	998	520	75
**PS/IRGANOX**	98.00/2.00	1099	520	75
**PS/IRGANOX**	99.00/1.00	1049	519	74

**PS/IRGANOX**	99.50/0.50	1050	519	74
**PS/IRGANOX**	99.75/0.25	1183	520	75
**PS/PV blue**	92.00/8.00	1117	482	37
**PS/PV blue**	94.00/4.00	2111	483	38
**PS/PV blue**	98.00/2.00	3383	484	39
**PS/PV blue**	99.00/1.00	3971	486	41
**PS/PV blue**	99.50/0.50	4745	488	43
**PS/PV blue**	99.75/0.25	3693	493	48
**PS/PV red**	92.00/8.00	868	620	175
**PS/PV red**	94.00/4.00	1065	618	173
**PS/PV red**	98.00/2.00	1446	617	172
**PS/PV red**	99.00/1.00	2303	612	167
**PS/PV red**	99.50/0.50	3510	606	161
**PS/PV red**	99.75/0.25	5339	523	78
**LDPE**	granulate	1317	526	81
**LDPE/$CaCO_3$**	60.00/40.00	5265	531	86
**LDPE/$CaCO_3$**	80.00/20.00	3047	524	79
**LDPE/$CaCO_3$**	90.00/10.00	1339	521	76
**LDPE/$CaCO_3$**	95.00/5.00	550	516	71
**LDPE/$CaCO_3$**	97.50/2.50	288	514	69
**LDPE/$CaCO_3$**	98.75/1.25	191	513	68
**LDPE/Talcum**	60.00/40.00	17782	488	43
**LDPE/Talcum**	80.00/20.00	7361	488	43
**LDPE/Talcum**	90.00/10.00	3923	490	45
**LDPE/Talcum**	95.00/5.00	1809	502	57
**LDPE/Talcum**	97.50/2.50	810	507	62
**LDPE/Talcum**	98.75/1.25	318	513	68
**LDPE/BYK**	92.00/8.00	537	520	75
**LDPE/BYK**	94.00/4.00	346	516	71
**LDPE/BYK**	98.00/2.00	212	514	69
**LDPE/BYK**	99.00/1.00	177	513	68
**LDPE/BYK**	99.50/0.50	126	512	67
**LDPE/BYK**	99.75/0.25	138	513	68
**LDPE/IRGAFOS**	92.00/8.00	621	516	71
**LDPE/IRGAFOS**	94.00/4.00	553	514	69
**LDPE/IRGAFOS**	98.00/2.00	562	514	69
**LDPE/IRGAFOS**	99.00/1.00	402	514	69
**LDPE/IRGAFOS**	99.50/0.50	344	514	69
**LDPE/IRGAFOS**	99.75/0.25	222	512	67
**LDPE/IRGANOX**	92.00/8.00	226	517	72
**LDPE/IRGANOX**	94.00/4.00	143	515	70
**LDPE/IRGANOX**	98.00/2.00	128	514	69
**LDPE/IRGANOX**	99.00/1.00	118	512	67
**LDPE/IRGANOX**	99.50/0.50	110	513	68
**LDPE/IRGANOX**	99.75/0.25	94	514	69
**LDPE/PV blue**	92.00/8.00	73	485	40
**LDPE/PV blue**	94.00/4.00	98	486	41
**LDPE/PV blue**	98.00/2.00	138	486	41
**LDPE/PV blue**	99.00/1.00	128	489	44
**LDPE/PV blue**	99.50/0.50	114	500	55

**LDPE/PV blue**	99.75/0.25	166	505	60
**LDPE/PV red**	92.00/8.00	971	621	176
**LDPE/PV red**	94.00/4.00	1053	620	175
**LDPE/PV red**	98.00/2.00	903	618	173
**LDPE/PV red**	99.00/1.00	884	615	170
**LDPE/PV red**	99.50/0.50	790	610	165
**LDPE/PV red**	99.75/0.25	621	600	155
**$CaCO_3$**	powder	480	543	98
**Talcum**	powder	2559	512	67
**BYK**	granule	3552	523	78
**IRGAFOS**	powder	602	519	74
**IRGANOX**	powder	606	519	74
**PV blue**	powder	21	900	455
**PV red**	powder	225	622	177

Table S2. Results from Gaussian analyzing of the phase-dependent fluorescence lifetime τ_{PH} $[ns]$ from ten FD-FLIM images per sample.

**Compound**	**Concentration wt%**	τ_{PH} $[ns]$
**PP**	IM	1.88 ± 0.06
**PP/Talcum**	60.00/40.00	2.09 ± 0.05
**PP/Talcum**	80.00/20.00	2.08 ± 0.07
**PP/Talcum**	90.00/10.00	2.07 ± 0.07
**PP/Talcum**	95.00/5.00	2.01 ± 0.08
**PP/Talcum**	97.50/2.50	1.91 ± 0.07
**PP/Talcum**	98.75/1.25	1.83 ±0.08
**PP/BYK**	92.00/8.00	2.32 ± 0.08
**PP/BYK**	94.00/4.00	2.26 ± 0.07
**PP/BYK**	98.00/2.00	2.14 ± 0.06
**PP/BYK**	99.00/1.00	2.08 ± 0.05
**PP/BYK**	99.50/0.50	2.05 ± 0.05
**PP/BYK**	99.75/0.25	2.01 ± 0.05
**PP/IRGAFOS**	92.00/8.00	2.76 ± 0.10
**PP/IRGAFOS**	94.00/4.00	2.41 ± 0.10
**PP/IRGAFOS**	98.00/2.00	2.20 ± 0.13
**PP/IRGAFOS**	99.00/1.00	2.07 ± 0.10
**PP/IRGAFOS**	99.50/0.50	2.09 ± 0.09
**PP/IRGAFOS**	99.75/0.25	2.02 ± 0.06
**PP/IRGANOX**	92.00/8.00	2.36 ± 0.09
**PP/IRGANOX**	94.00/4.00	2.20 ± 0.06
**PP/IRGANOX**	98.00/2.00	2.07 ± 0.06

**PP/IRGANOX**	99.00/1.00	2.06 ± 0.06
**PP/IRGANOX**	99.50/0.50	2.00 ± 0.05
**PP/IRGANOX**	99.75/0.25	1.98 ± 0.05
**PP/PV blue**	92.00/8.00	0.14 ± 0.03
**PP/PV blue**	94.00/4.00	0.16 ± 0.03
**PP/PV blue**	98.00/2.00	0.23 ± 0.06
**PP/PV blue**	99.00/1.00	0.73 ± 0.17
**PP/PV blue**	99.50/0.50	0.91 ± 0.13
**PP/PV blue**	99.75/0.25	1.20 ± 0.09
**PP/PV red**	92.00/8.00	0.28 ± 0.07
**PP/PV red**	94.00/4.00	0.31 ± 0.07
**PP/PV red**	98.00/2.00	0.30 ± 0.08
**PP/PV red**	99.00/1.00	0.33 ± 0.08
**PP/PV red**	99.50/0.50	0.39 ± 0.09
**PP/PV red**	99.75/0.25	0.66 ± 0.10
**PS**	granulate	2.01 ± 0.06
**PS/$CaCO_3$**	60.00/40.00	2.69 ± 0.06
**PS/$CaCO_3$**	80.00/20.00	2.67 ± 0.08
**PS/$CaCO_3$**	90.00/10.00	2.60 ± 0.08
**PS/$CaCO_3$**	95.00/5.00	2.44 ± 0.09
**PS/$CaCO_3$**	97.50/2.50	2.58 ± 0.07
**PS/$CaCO_3$**	98.75/1.25	1.99 ± 0.11
**PS/Talcum**	60.00/40.00	2.41 ± 0.04
**PS/Talcum**	80.00/20.00	2.42 ± 0.04
**PS/Talcum**	90.00/10.00	2.40 ± 0.05
**PS/Talcum**	95.00/5.00	2.35 ± 0.05
**PS/Talcum**	97.50/2.50	2.26 ± 0.06
**PS/Talcum**	98.75/1.25	2.18 ± 0.07
**PS/BYK**	92.00/8.00	3.13 ± 0.09
**PS/BYK**	94.00/4.00	3.05 ± 0.09
**PS/BYK**	98.00/2.00	2.69 ± 0.10
**PS/BYK**	99.00/1.00	2.36 ± 0.10
**PS/BYK**	99.50/0.50	2.34 ± 0.11
**PS/BYK**	99.75/0.25	2.20 ± 0.10
**PS/IRGAFOS**	92.00/8.00	2.85 ± 0.08
**PS/IRGAFOS**	94.00/4.00	2.73 ± 0.08
**PS/IRGAFOS**	98.00/2.00	2.71 ± 0.08
**PS/IRGAFOS**	99.00/1.00	2.74 ± 0.07
**PS/IRGAFOS**	99.50/0.50	2.68 ± 0.07
**PS/IRGAFOS**	99.75/0.25	2.35 ± 0.09
**PS/IRGANOX**	92.00/8.00	1.92 ± 0.07
**PS/IRGANOX**	94.00/4.00	1.76 ± 0.08
**PS/IRGANOX**	98.00/2.00	1.75 ± 0.09
**PS/IRGANOX**	99.00/1.00	1.81 ± 0.08
**PS/IRGANOX**	99.50/0.50	1.99 ± 0.07
**PS/IRGANOX**	99.75/0.25	2.27 ± 0.07
**PS/PV blue**	92.00/8.00	0.93 ± 0.06

**PS/PV blue**	94.00/4.00	1.02 ± 0.05
**PS/PV blue**	98.00/2.00	1.11 ± 0.05
**PS/PV blue**	99.00/1.00	1.17 ± 0.05
**PS/PV blue**	99.50/0.50	1.27 ± 0.04
**PS/PV blue**	99.75/0.25	1.42 ± 0.05
**PS/PV red**	92.00/8.00	1.13 ± 0.10
**PS/PV red**	94.00/4.00	1.77 ± 0.09
**PS/PV red**	98.00/2.00	2.50 ± 0.09
**PS/PV red**	99.00/1.00	3.28 ± 0.09
**PS/PV red**	99.50/0.50	3.80 ± 0.09
**PS/PV red**	99.75/0.25	4.18 ± 0.07
**LDPE**	granulate	3.94 ± 0.21
**LDPE/$CaCO_3$**	60.00/40.00	2.80 ± 0.07
**LDPE/$CaCO_3$**	80.00/20.00	2.84 ± 0.08
**LDPE/$CaCO_3$**	90.00/10.00	2.79 ± 0.10
**LDPE/$CaCO_3$**	95.00/5.00	2.34 ± 0.12
**LDPE/$CaCO_3$**	97.50/2.50	1.90 ± 0.18
**LDPE/$CaCO_3$**	98.75/1.25	1.25 ± 0.20
**LDPE/Talcum**	60.00/40.00	2.42 ± 0.04
**LDPE/Talcum**	80.00/20.00	2.31 ± 0.06
**LDPE/Talcum**	90.00/10.00	2.31 ± 0.06
**LDPE/Talcum**	95.00/5.00	2.24 ± 0.07
**LDPE/Talcum**	97.50/2.50	2.03 ± 0.09
**LDPE/Talcum**	98.75/1.25	1.66 ± 0.15
**LDPE/BYK**	92.00/8.00	2.45 ± 0.13
**LDPE/BYK**	94.00/4.00	2.15 ± 0.16
**LDPE/BYK**	98.00/2.00	1.48 ± 0.21
**LDPE/BYK**	99.00/1.00	1.21 ± 0.21
**LDPE/BYK**	99.50/0.50	0.78 ± 0.23
**LDPE/BYK**	99.75/0.25	0.64 ± 0.23
**LDPE/IRGAFOS**	92.00/8.00	2.45 ± 0.13
**LDPE/IRGAFOS**	94.00/4.00	2.31 ± 0.13
**LDPE/IRGAFOS**	98.00/2.00	2.11 ± 0.12
**LDPE/IRGAFOS**	99.00/1.00	1.74 ± 0.12
**LDPE/IRGAFOS**	99.50/0.50	1.57 ± 0.13
**LDPE/IRGAFOS**	99.75/0.25	1.28 ± 0.14
**LDPE/IRGANOX**	92.00/8.00	1.15 ± 0.16
**LDPE/IRGANOX**	94.00/4.00	0.82 ± 0.20
**LDPE/IRGANOX**	98.00/2.00	0.61 ± 0.20
**LDPE/IRGANOX**	99.00/1.00	0.40 ± 0.21
**LDPE/IRGANOX**	99.50/0.50	0.30 ± 0.11
**LDPE/IRGANOX**	99.75/0.25	0.24 ± 0.06
**LDPE/PV blue**	92.00/8.00	0.20 ± 0.04
**LDPE/PV blue**	94.00/4.00	0.45 ± 0.11
**LDPE/PV blue**	98.00/2.00	0.81 ± 0.28
**LDPE/PV blue**	99.00/1.00	0.93 ± 0.25
**LDPE/PV blue**	99.50/0.50	1.04 ± 0.24

**LDPE/PV blue**	99.75/0.25	1.08 ± 0.23
**LDPE/PV red**	92.00/8.00	0.31 ± 0.07
**LDPE/PV red**	94.00/4.00	0.29 ± 0.08
**LDPE/PV red**	98.00/2.00	0.29 ± 0.08
**LDPE/PV red**	99.00/1.00	0.29 ± 0.09
**LDPE/PV red**	99.50/0.50	0.32 ± 0.10
**LDPE/PV red**	99.75/0.25	0.33 ± 0.11
**$CaCO_3$**	powder	1.95 ± 0.23
**Talcum**	powder	2.25 ± 0.09
**BYK**	granule	2.78 ± 0.07
**IRGAFOS**	powder	1.99 ± 0.17
**IRGANOX**	powder	2.36 ± 0.12
**PV blue**	powder	--
**PV red**	powder	0.10 ± 0.01

Fluorescence Lifetime Models:

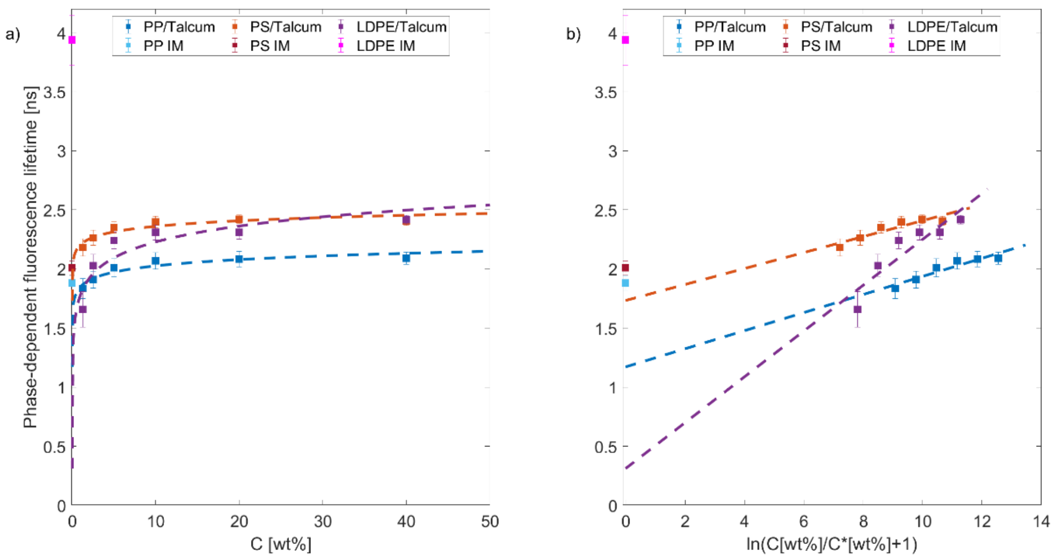

Figure S22: a) Dashed lines show the logarithmic fit from equation (4) onto the measured fluorescence lifetimes of the composites PP/Talcum, PS/Talcum and LDPE/Talcum. The fluorescence lifetimes of the pure IM plastics are displayed at 0% concentration. b) Shows the fits in a logarithmic x scaling, whereby it can clearly be seen, that the fluorescence lifetimes of the pure IM materials cannot be related to the behavior of the fluorescence lifetimes of the composites.

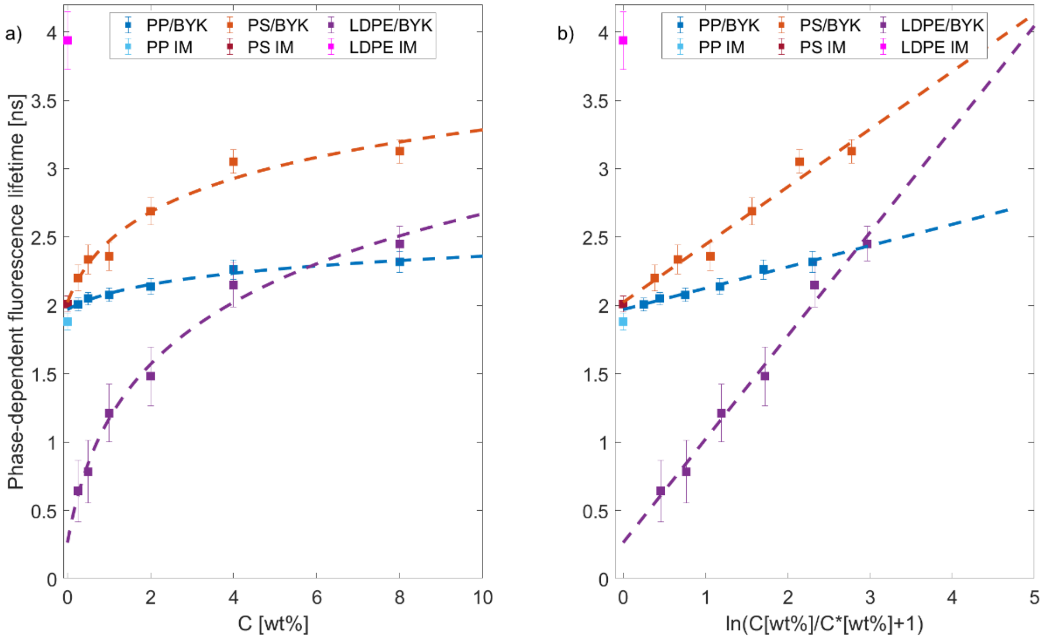

Figure S23. a) Dashed lines show the logarithmic fit from equation (4) onto the measured fluorescence lifetimes of the composites PP/BYK, PS/BYK and LDPE/BYK. The fluorescence lifetimes of the pure IM plastics are displayed at 0% concentration. b) Shows the fits in a logarithmic x scaling, whereby it can clearly be seen, that the fluorescence lifetimes of the pure IM materials cannot be related to the behavior of the fluorescence lifetimes of the composites.

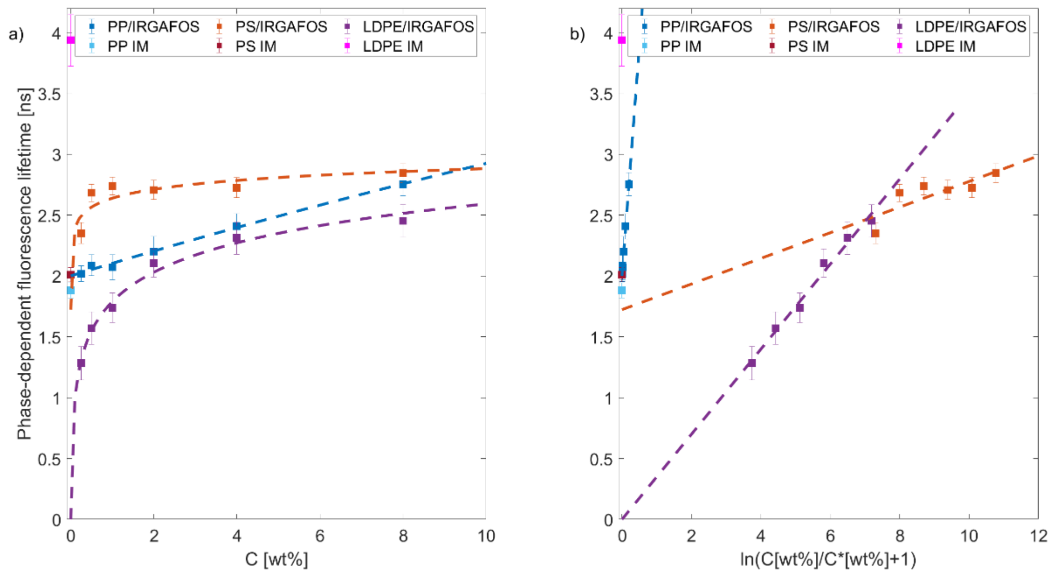

Figure S24. a) Dashed lines show the logarithmic fit from equation (4) onto the measured fluorescence lifetimes of the composites PP/IRGAFOS, PS/IRGAFOS and LDPE/IRGAFOS. The fluorescence lifetimes of the pure IM plastics are displayed at 0% concentration. b) Shows the fits in a logarithmic x scaling, whereby it can clearly be seen, that the fluorescence lifetimes of the pure IM materials cannot be related to the behavior of the fluorescence lifetimes of the composites.

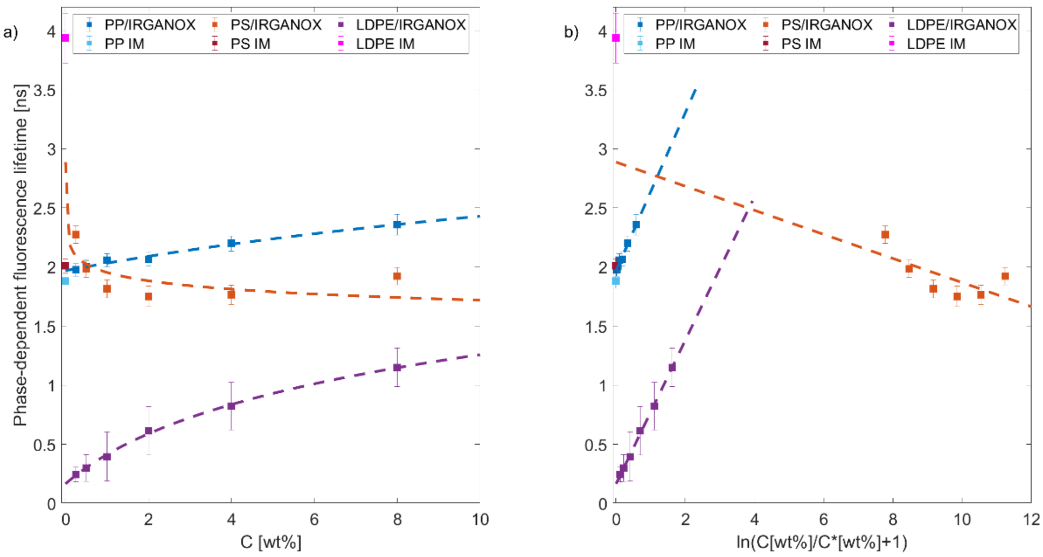

Figure S25. a) Dashed lines show the logarithmic fit from equation (4) onto the measured fluorescence lifetimes of the composites PP/IRGANOX, PS/IRGANOX and LDPE/IRGANOX. The fluorescence lifetimes of the pure IM plastics are displayed at 0% concentration. b) Shows the fits in a logarithmic x scaling, whereby it can clearly be seen, that the fluorescence lifetimes of the pure IM materials cannot be related to the behavior of the fluorescence lifetimes of the composites.

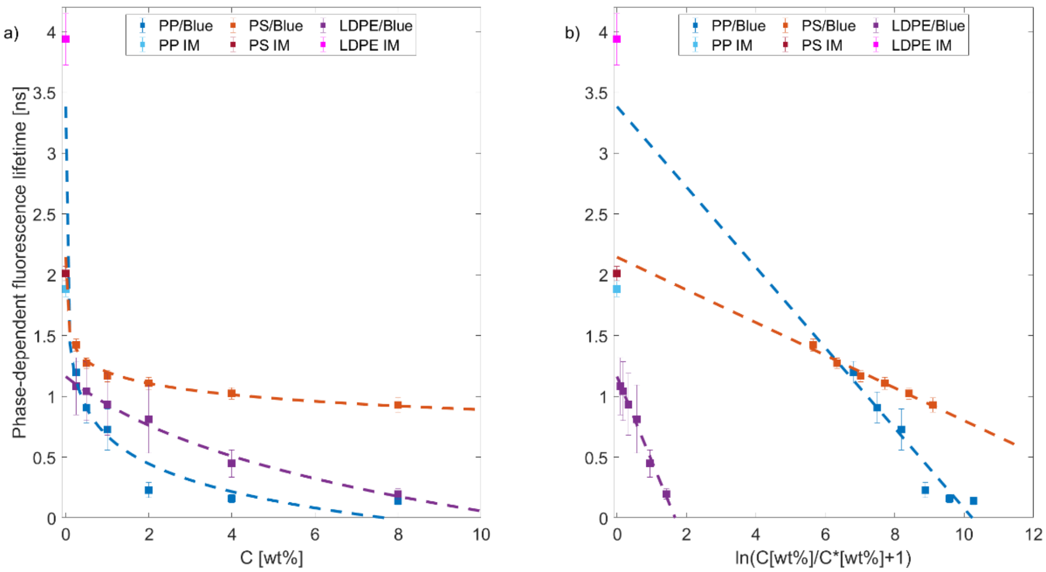

Figure S26. a) Dashed lines show the logarithmic fit from equation (4) onto the measured fluorescence lifetimes of the composites PP/Blue, PS/Blue and LDPE/Blue. The fluorescence lifetimes of the pure IM plastics are displayed at 0% concentration. b) Shows the fits in a logarithmic x scaling, whereby it can clearly be seen, that the fluorescence lifetimes of the pure IM materials cannot be related to the behavior of the fluorescence lifetimes of the composites.

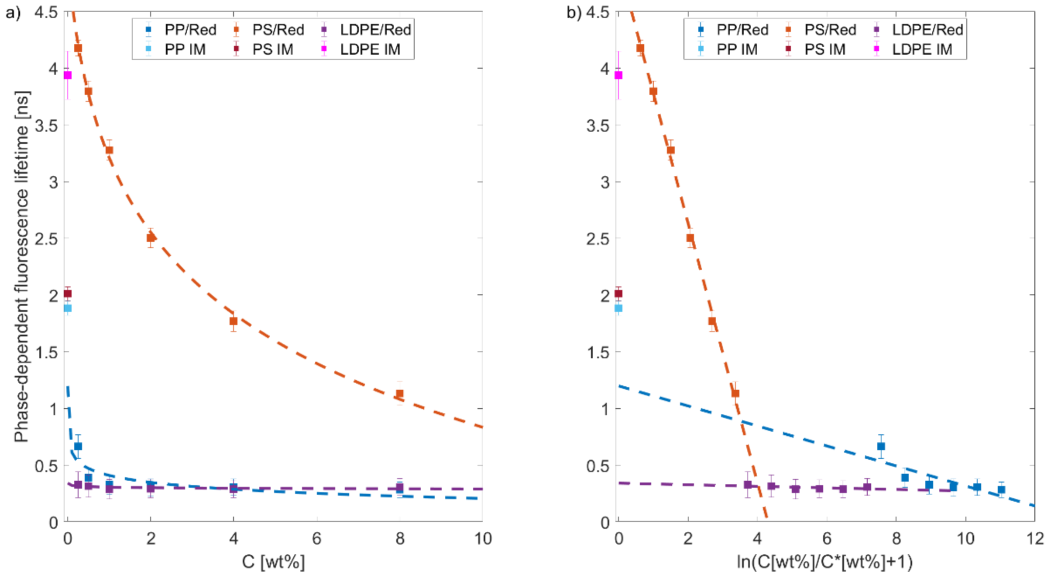

Figure S27. a) Dashed lines show the logarithmic fit from equation (4) onto the measured fluorescence lifetimes of the composites PP/Red, PS/Red and LDPE/Red. The fluorescence lifetimes of the pure IM plastics are displayed at 0% concentration. b) Shows the fits in a logarithmic x scaling, whereby it can clearly be seen, that the fluorescence lifetimes of the pure IM materials cannot be related to the behavior of the fluorescence lifetimes of the composites.
